# Label‐Free Super‐Resolution Imaging Using Non‐Linear Exogenous Photon Avalanche Backlight

**DOI:** 10.1002/advs.78676

**Published:** 2026-11-09

**Authors:** S. Karmegam, M. Szalkowski, M. Misiak, K. Prorok, D. Szymański, A. Bednarkiewicz

**Affiliations:** ^1^ Institute of Low Temperature and Structure Research Polish Academy of Sciences Wrocław Poland; ^2^ Nanophotonics Group Institute of Physics Faculty of Physics, Astronomy and Informatics Nicolaus Copernicus University in Toruń Toruń Poland; ^3^ Institute of Advanced Studies Nicolaus Copernicus University in Toruń Poland

**Keywords:** backlight, diffraction, imaging tool, luminescence, materials science, microscopic imaging, microscopy, nanophotonics, optics, optoelectronics

## Abstract

Optical imaging beyond the diffraction limit of light enables direct investigation of the structure, organization and functioning of sub‐micrometer, highly transparent objects such as subcellular components, thin films and nano‐engineered devices. However, most existing super‐resolution optical techniques rely on fluorescent labels, which can alter sample properties, suffer from phototoxicity and photobleaching, and require complex instrumentation. At the same time, alternative label‐free super‐resolution methods remain technically complex, demanding, costly and often difficult to implement. Here we propose and experimentally demonstrate a new concept of label‐free super‐resolution optical imaging termed transmission avalanche backlight (TAB) microscopy. TAB microscopy exploits strong optical nonlinearities in exogenous photon‐avalanche materials, which act as a far‐field created, luminescent backlight forming a near‐field virtual, fluorescent nano‐aperture. Weak attenuation and scattering by highly transparent samples are translated into strongly amplified luminescence modulation of the avalanche backlight substrate, resulting in enhanced image contrast. Using a single‐beam laser‐scanning configuration, TAB microscopy achieves far‐field, reconstruction‐free imaging with optical resolution of approximately 180 nm. This approach provides a relatively technically simple, label‐free route to sub‐diffraction imaging and may complement existing techniques across biology, materials science, nanophotonics and nanoengineering.

## Graphical abstract

Transmission avalanche backlight (TAB) microscopy turns a photon‐avalanching Tm^3^^+^:LiYF_4_ crystal into a nonlinear luminescent backlight. Weak attenuation by a transparent sample is amplified into strong emission modulation: image contrast increases with sample attenuation and avalanche nonlinearity (*N*), while the effective point spread function narrows as 1/√*N*, enabling label‐free, reconstruction‐free sub‐diffraction imaging with a single scanned near‐infrared beam.

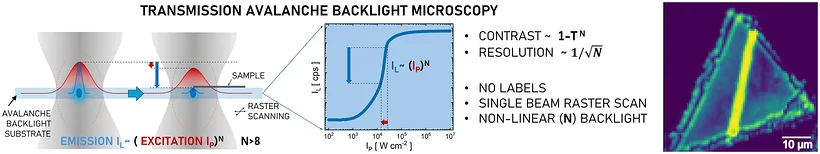

## 1 Introduction

Current progress and understanding in (micro)biology [1, 2, 3], physiology [4, 5, 6], physics [7] or technology [8] are often possible thanks to the ability to in situ, in vitro or in vivo visualize the morphology, appearance, functioning and behavior of submicron or nanometer objects, preferably using non‐destructive light and optical methods. These advancements became possible thanks to developing groundbreaking concepts of optical super‐resolution imaging (SRI), together with dedicated instruments and sophisticated data acquisition or analysis (Tables S1 and S2) [9, 10, 11, 12, 13]. However, most of these techniques depend on fluorescent labeling [14, 15, 16, 17] of the delicate samples, necessitating the development of specialized fluorescent labels to balance target specificity, reduced (photo)toxicity and suitable photophysical and chemical properties [18]. To mitigate issues such as photobleaching, phototoxicity, spectral bleed‐through and limited multiplexing capability of conventional fluorophores, new classes of luminescent nanoparticle (NP)‐based labels have also been developed. These labels offer several advantages, including enhanced photostability, blinking‐free emission and background‐free detection, for example through efficient upconversion of near‐infrared (NIR) excitation into the visible or NIR spectral range [19, 20]. Although many challenges associated with biofunctionalization, target specificity, multiplexing and sensitivity of such fluorescent label‐based super‐resolution imaging (SRI) methods have been successfully addressed, the incorporation of any exogenous labels in general—whether molecular or NP‐based, may unfortunately perturb the delicate machinery and activity of living cells or alter the intrinsic properties of photonic devices under investigation. As a result, fluorescent imaging often provides only a static snapshot of a perturbed system, rather than enabling prolonged, remote and non‐invasive observation of the native sample. In addition, many fluorescence based SRI optical techniques remain complex and cumbersome to implement, owing to sophisticated optical instrument design [21, 22, 23] or to tedious fluorescence image acquisition, processing and interpretation [11, 24, 25]. Although several alternative SRI techniques have been proposed—such as plasmonic structured illumination microscopy (SIM) [26, 27], hyperbolic metamaterials based SIM [28, 29], and solid immersion lens approach [30, 31], they do not overcome all limitations of existing methods. When remote optical readout is not required, SRI may be also achieved with atomic force microscopy (AFM) or near‐field techniques, such as scanning near‐field optical microscopy (SNOM) or tip‐enhanced near‐field optical microscopy (TENOM). However, the applicability of these methods is largely restricted to those nanoobjects, which allow for direct physical contact between the probe tip and the sample surface. In addition, numerous technical difficulties—such as probe contamination and insufficient image quality, have so far hindered wider adoption, particularly in biology. Consequently, the development of novel, label‐free, non‐destructive, technically affordable and direct (reconstruction‐free) imaging techniques for in situ visualization of highly transparent samples below diffraction limit of light remains both highly desirable and challenging.

To circumvent alterations of the properties or dynamics of biological or photonic samples caused by incorporation of exogenous fluorescent labels [32, 33, 34], while still retaining access to super‐resolution optical imaging, several label‐free approaches have been proposed [35, 36]. The Abbe–Rayleigh diffraction limit can be surpassed by generating super oscillatory wavefronts [37, 38], by super oscillatory lenses (SOL) and SOL microscopes [39, 40], coherent scattering under oblique angle illumination [41], phase SIM [42], expansion stimulated Raman vibrational imaging [43], photon‐reassignment [44] or reconstruction topological microscopy [39]. In addition, the stimulated emission depletion (STED) approach—one of the current gold standards in fluorescence microscopy, can be adapted to label‐free imaging by exploiting the intrinsic saturable absorption of the sample [45]. Other methods rely on photothermal gradient effects in the sample [46], graphene quenched backlight Pr^3+^ fluorescence [47] or pump‐probe imaging of endogenous chromophores [45]. Furthermore, femtosecond stimulated Raman spectroscopy has been employed for label‐free identification of vibrational fingerprints of chemical species or functional groups [48] at spatial resolutions beyond the diffraction limit. Despite demonstrating genuine resolution enhancement without the use of exogenous fluorescent labels [35, 36], the majority of these methods suffer from one or more drawbacks, including instrumental complexity, cost inefficiency, near‐field detection requirements, computationally intensive image reconstruction with a priori knowledge on illumination pattern or sample properties, or the need for sophisticated, custom‐designed optical components such as lenses, metamaterials or plasmonic layers. As a result, many of these approaches remain difficult to implement for routine imaging of biological or highly transparent samples. A potential route to tackle these limitations lies in radically new imaging concepts that exploit unconventional materials, such as highly nonlinear photon avalanching (PA) bulk crystal substrates.

The photon avalanche (PA) phenomenon was discovered in 1979 in bulk LaCl_3_:Pr^3+^ single crystals [49] and was initially exploited for mid‐infrared (MIR) photon detection. For over the subsequent four decades, only a limited number of studies reported PA in other lanthanides (Ho, Tm, Sm, Nd, Er) doped bulk materials (single crystals, waveguides, glasses) predominantly in cryogenic temperatures (4.2–77 K), and primarily for MIR photons detection or upconversion‐based lasers [50, 51, 52]. Photon avalanching relies on a positive loop involving sequential excited state absorption (ESA) and energy cross‐relaxation (CR) between neighbor lanthanide ions (Figure 1a), which, above a distinct excitation threshold, gives rise to an extremely steep dependence of luminescence intensity (*I*_L_) on pump power(*I*_P_), following power law *I*_L_ = (*I*_P_)^*N*^. The corresponding nonlinearity index *N* can exceed values of 10–20, typically reaching 20–40 [53, 54, 55, 56] and, in exception cases approaching 500 [57] (Figure 1b). Only recently PA has been demonstrated in nano‐ and micro‐sized inorganic materials co‐doped with selected lanthanide ions [53, 54, 56]. Owing to their extreme optical non‐linearity and complex photoluminescence kinetic, these materials have enabled a range of emerging applications, including single beam sub‐diffraction imaging (PASSI) (Chapter S1.5) [53, 54, 56], optical photo‐switching and sub‐nanometer localization microscopy [58], as well as reservoir computing [59], pico‐newton force sensing [60] and temperature sensing [61]. Notably, direct far‐field super‐resolution imaging with spatial resolution down to *λ*/5 (≈70 nm for *S* = 23) has been proposed [62] and experimentally demonstrated [53] using highly non‐linear 800 nm emission of thulium (Tm^3+^) doped inorganic photon avalanching nanoparticles (ANPs) under 1064 nm excitation. Importantly, the strong nonlinearity of these nano emitters allows ANP‐based SRI to be implemented using technically simple and cost‐effective confocal laser‐scanning microscopes, requiring only minor modifications such as a customized filter cube and a dedicated, cheap NIR excitation source. Nevertheless, despite these advances, such approaches still rely on the incorporation of (biofunctionalized) photon‐avalanche nanoparticle labels into the imaged specimen.

**FIGURE 1:**
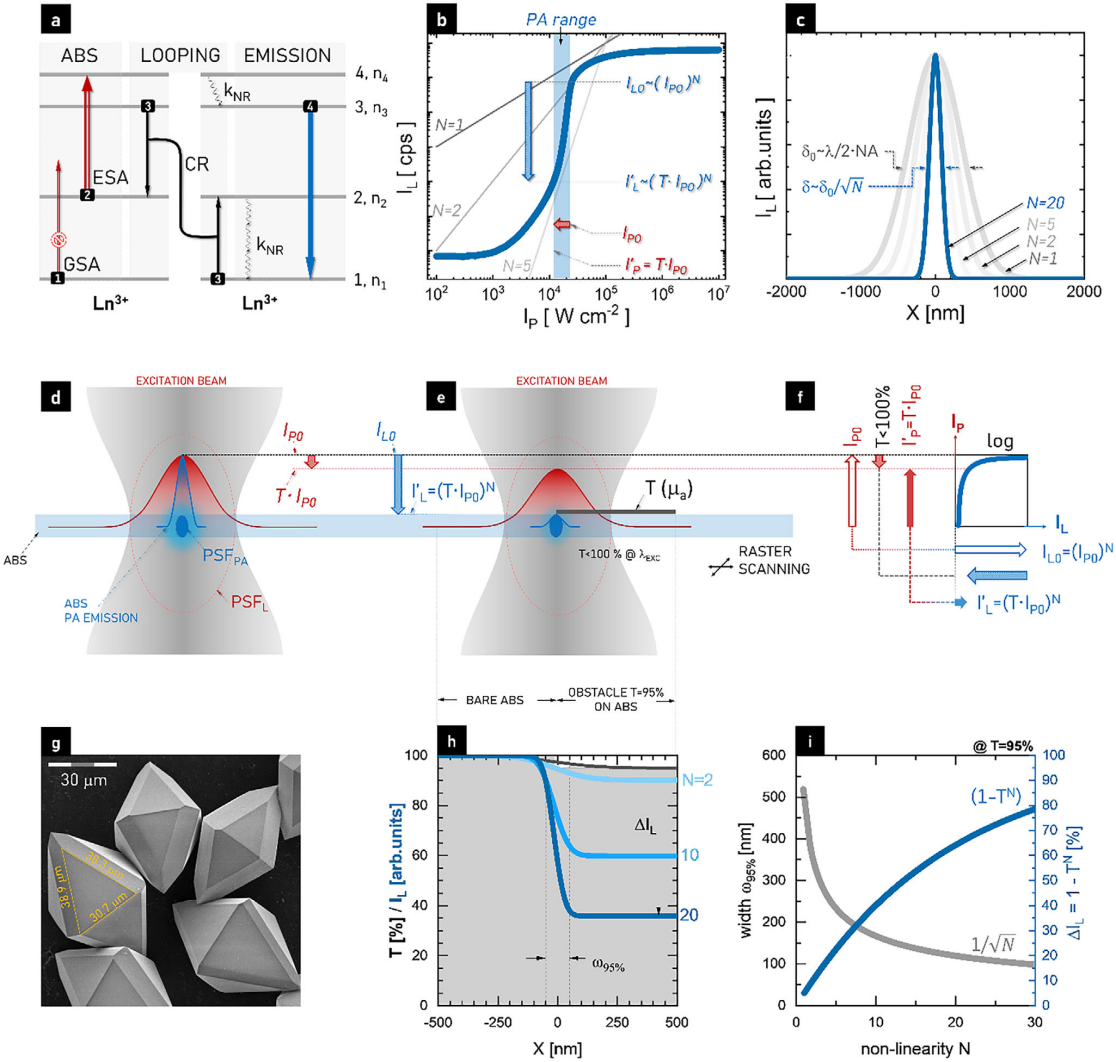
The principles behind photon avalanche (PA) and transmission avalanche backlight microscopy (TAB). (a) General mechanisms leading to photon avalanche emission—weak GSA (1), efficient ESA resonant absorption (2) and energy cross‐relaxation (CR) based energy looping (3) leads to (b) highly non‐linear emission (4) intensity dependence versus pump power intensity (PPD); as a reference, linear (*N* = 1) and non‐linear (*N* = 2 and 5) PPD are included. (c) Cross section of a diffraction limited gaussian beam for a linear PDD (*N* = 1) and effective, luminescence cross‐section for nonlinear (*N* = 2,5,20) PA materials; (d) the effective point spread function (PSF_PA_) width is reduced by a factor of $\sqrt{N}$ as compared to a linear case (PSF_L_); (e) a semi‐translucent obstacle (*T* < 100%) reduces excitation beam intensity to *T* · *I*_P0_, and this small decrease is translated to huge PA emission intensity decrease by a factor of *T^N^*, from the original *I*_L0_ = (*I*_P0_)^*N*^ to *I*′_L_ = (*T* · *I*_P0_)^*N*^. (f) Explanation of how the photoexcitation intensity (red arrows) upon presence of obstacle (*T* < 100%) is converted to luminescence intensity (blue arrows) by the non‐linear photon avalanche backlight substrate; the empty, dashed and solid arrows indicate original (without obstacle), relative change and effective (with obstacle) signal intensities, respectively. (g) Representative SEM image of photon avalanching 3%Tm^3+^ doped LiYF_4_ ABS microcrystals, the foot area of this crystal equals ca. 540 µm^2^. (h) Simulation of PA luminescence intensity of the ABS across the semitransparent obstacle (*T* = 95%, panel e) for a linear (*N* = 1, gray) and non‐linear (*N* = 2, 10 and 20, blue profiles) ABS crystals; (i) the simulations of non‐linearity *N* dependent width ω_95%_ (left) and PA luminescence intensity change (right); the ω_95%_ and $\Delta I_{L}$ are defined graphically on panel (h); the ω_95%_ indicate the edge width (in nanometers) corresponding the intensity loss from 5% to 95% at the semi‐translucent layer edge; the $\Delta I_{L}$ is the non‐linear PA intensity loss due to sample of 95% transmission as compared to pristine PA substrate emission intensity.

In contrast to PASSI and other conventional fluorescent label‐based SRI approaches, here we propose, simulate and experimentally demonstrate a fundamentally different SRI concept termed transmission avalanche backlight (TAB) microscopy (Figure 1). TAB microscopy exploits the same extreme optical nonlinearity luminescent properties of avalanching materials (Figure 1c); however, instead of embedding the nonlinearity within endogenous nano‐labels, it is provided by a bulk photon‐avalanche crystal that acts as a large‐area, exogenous homogenous avalanche backlight substrate (ABS). The pristine, unlabeled sample is placed directly on top of the substrate and imaged without any chemical or structural modification. Upon photoexcitation of the nonlinear ABS with a diffraction‐limited laser beam of characteristic width σ_0_, the effective fluorescence emission region becomes spatially confined to $\sigma_{0}/\sqrt{N}$ owing to the extreme nonlinearity of the PA process (see Chapter S2). This confinement is conceptually analogous to near‐field scanning of the fluorescent sample with the subwavelength aperture in the SNOM tip, yet here the aperture is not physical hole but is instead formed remotely within the ABS by far‐field excitation. We therefore introduce the concept of a virtual aperture (VA), generated inside an ideally extended, flat and thin avalanche substrate, eliminating the need for a physical scanning tip. Simultaneously, minute spatial fluctuations of the excitation intensities beneath the confined VA, arising from attenuation, scattering or energy‐transfer interaction within the semi‐transparent sample are translated by the photon‐avalanche nonlinearity into strongly amplified photoluminescence modulations, resulting in substantial contrast enhancement (Figure 1d). Because the virtual aperture is smaller than the diffraction limited excitation spot (Figure S1), TAB microscopy enables optical super‐resolution beyond the diffraction limit, analogously to SNOM (Table S1). Crucially, however, unlike SNOM, where a physical aperture scans the sample's surface, the VA in TAB microscopy scans the sample throughout its volume along the optical path of the laser, without mechanical contact or probe‐induced perturbation. As no exogenous labels are required, image formation relies exclusively on intrinsic light‐matter interactions between the focused beam and the nano‐structured and/or highly transparent sample (Figure 1d,e). Although these interactions occur in the near field at the sample‐substrate interface, the spatially confined PA emission of the ABS is generated and directly detected in far‐field through fluorescence readout. Consequently, by relocating extreme photon‐avalanche nonlinearity from exogenous nano‐labels into a macroscopic backlight substrate, TAB microscopy provides reconstruction‐free, far‐field super‐resolution imaging with enhanced contrast and sub‐diffraction spatial resolution, while fully preserving the native state of the investigated sample.

In the newly proposed TAB microscopy concept, rather than measuring directly the incident or transmitted photon flux, the light transmitted through the specimen is used to photoexcite luminescence of the highly non‐linear photon avalanching backlight substrate (Figure 1c). As a result, a highly—but not perfectly—translucent sample composed of single cells, nanostructures or thin layers partially attenuates the excitation beam before it reaches the exogenous ABS (Figure 1e vs d). Consequently, the effective excitation intensity incident on the ABS (Figure 1f, red solid arrow) is slightly reduced relative to the pristine case (red hashed arrow). Owing to the extreme nonlinearity of photon avalanche process (Figure 1b), this small decrease in excitation power is translated to disproportionally large reduction of the PA emission intensity (Figure 1f, blue solid arrow) compared with the emission from bare ABS (Figure 1f, blue hashed arrow). Therefore, without endogenous labeling the sample itself, TAB microscopy converts weak attenuation caused by physical obstacles along the optical path into a strongly amplified luminescence contrast of the PA substrate, enabling quantitative assessment of sample transmittance at the pumping wavelength. Quantitative evaluation can be performed using parameters defined in Methods section, including luminescence intensity ratio (LIR) (Equation 1), contrast (C) (Equation 2), sensitivity (Equation 3) or edge width ω_95%_ (presented in Figure 1h,i). For example, a sample exhibiting 95% transmission reduces the PA emission intensity to approximately 36% of its original value for an ABS with a nonlinearity index of 20 (Figure 1h,i). This in turn improves the contrast and enables visualization of such highly transparent heterogenous specimens with greater details. Furthermore, numerical simulations indicate that edges of thin layers can be resolved with improved optical resolution, following *N^−^* ^0.5^ scaling rule of thumb [62], reaching optical resolutions down to 100 nm for nonlinearities of *N* = 30.

The physical origin of the augmented optical resolution in TAB microscopy can be easily derived from analytical expressions (Chapter S2) but is less straightforward to capture the concept behind, although it originates from exactly the same effects as the one standing behind PASSI resolution enhancement [62]. When the diffraction‐limited excitation beam encounters an obstacle (Figure 1e) of decreased transparency T, the photon avalanche emission from the ABS decreases sharply as the beam is displaced from a non‐attenuated region into the semitransparent area. In contrast to a linear backlight configuration, where the excitation power—and consequently the fluorescence signal, scales proportionally with the transmittance (≈ *T* ^1^), the photon‐avalanche response follows a highly nonlinear dependence (*T^N^*). As a consequence, even small spatial variations in transmittance give rise to disproportionately large changes in emission intensity, effectively sharpening the spatial response of the system. This behavior can be interpreted as a reduction of the effective point spread function (PSF) volume in non‐linear avalanching substrate, which scales with an inverse square root of the nonlinearity index *N* (Figure 1i). To further elucidate and visualize these effects, we employed the same numerical framework (described shortly in TAB simulations section) previously developed to introduce the concept on super‐resolution imaging with non‐linear labels [53, 62]. Using this approach, we simulated the response of the ABS substrate to attenuating layers and particles over a broad range of nonlinearities (Figure 2, TAB simulations chapter S2).

**FIGURE 2:**
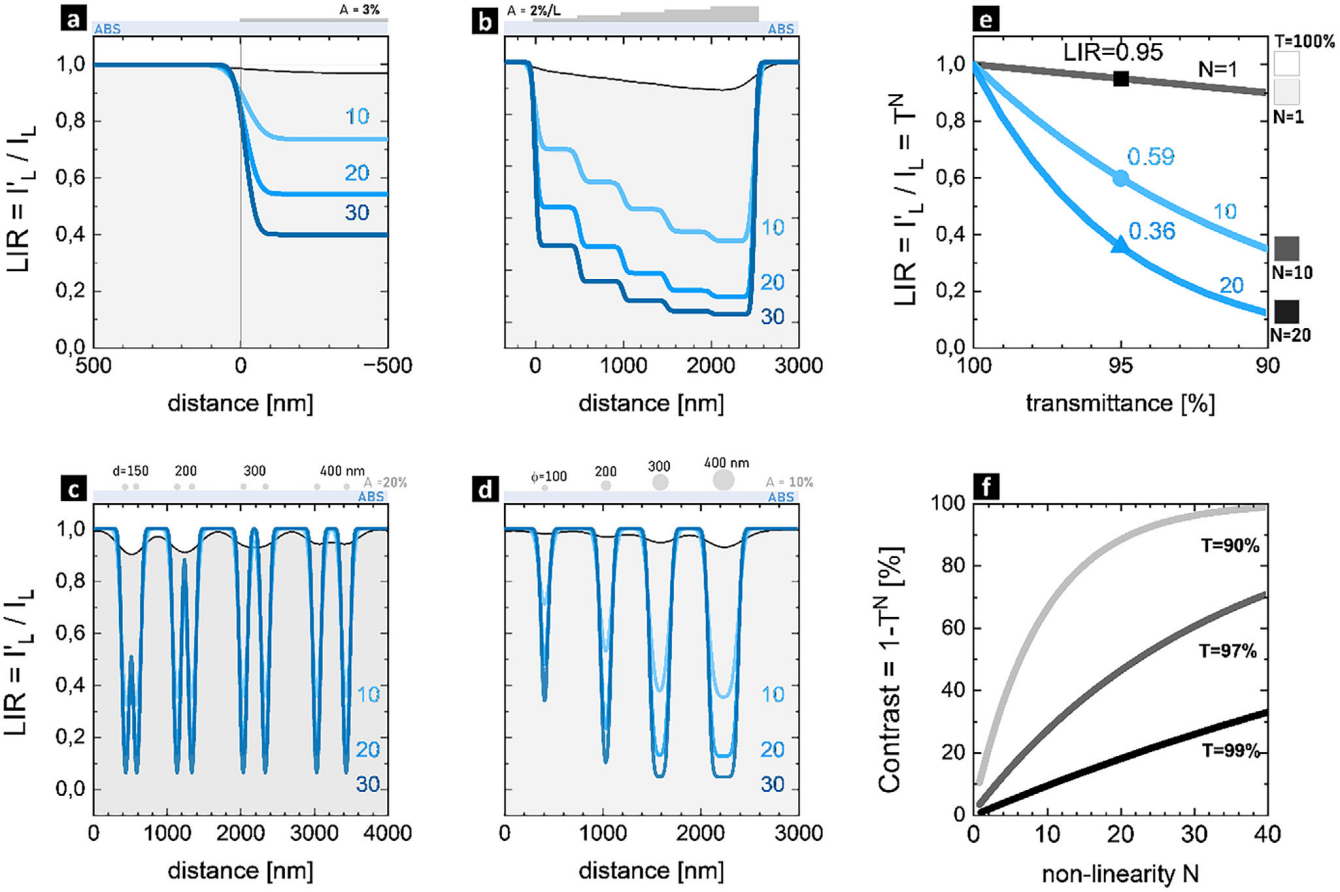
Simulations of TAB imaging with various samples types and rising non‐linearity index. Cross‐section of the TAB image across the (a) thin layer with 3% attenuation, (b) multiple thin overlapping layers with attenuation of 2% each, (c) two nanoparticles with attenuation of 20% separated by various distances (d = 150, 200, 300, 400 nm), (d) nanoparticles with attenuation of 10% and rising size (*ϕ* = 100, 200, 300, 400 nm). Black line—image cross section accounting for linear attenuation, blue lines—image cross‐sections accounting for avalanching substrate with rising nonlinearity index (*N* = 10, 20 and 30). (e) The LIR versus transmittance of the obstacle for ABS exhibiting a linear (gray) and non‐linearity index *N* = 10 and 20; On the right, the rectangles are exemplification of the PA brightness achieved through an obstacle with T = 90% for *N* = 1,10 and 20 (the white corresponds to maximum brightness); (f) the contrast *C = (1‐T^N^)* versus rising non‐linearity for obstacles exhibiting *T* = 90, 97 and 99%.

As expected, both contrast enhancement and augmented optical resolution scale with the non‐linearity index and manifest clearly in PA intensity cross‐sections for thin layers of varying attenuation (Figure 2a) or thickness (Figure 2b), ensembles of nanoparticles (Figure 2c) as well as individual nanoparticles of different size (Figure 2d). For clarity and computational efficiency, the simulations assume perfectly homogenous avalanching backlighting of the sample and neglect secondary optical effects such as light scattering, multiple reflections or light diffraction effects within the sample. While these simplifying assumptions allow isolation of the core TAB contrast and resolution mechanisms, such effects may become relevant under realistic experimental conditions and should be considered when interpreting TAB images. The effects observed in Figure 2a–d can be generalized, and displayed as the dependence of luminescence intensity ratio LIR defined with Equation 1 versus sample transmittance for various non‐linearity index N (Figure 2e), or as the dependence of contrast enhancement defined by Equation 2 versus the non‐linearity index N for various sample transmittance (Figure 2f).

To validate the TAB microscopy concept, we have first synthesized bulk ABS crystals doped with Tm^3+^ ions to purposefully augment energy cross‐relaxation and energy looping required to induce photon avalanche behavior. The Tm^3+^ doped LiYF_4_ crystals (Figure 1g) were made using hydrothermal synthesis method (synthesis of 3%Tm LiYF_4_ microcrystals chapter). The resulting crystals exhibited well‐defined reproducible morphology with relatively large lateral size, although moderate crystal‐to‐crystal size variation (Chapter S3) and negligible spatial variation of PA properties within single ABS crystal were observed (Chapter S3.2). The ABS crystals exhibited a sufficiently high nonlinearity index of 8.5 ± 1.0, together with moderate PA thresholds in the range of 11–16 kW cm^−2^ (Figure S8). Importantly, these ABS‐es showed no evidence of photodarkening—an effect previously reported for NaYF_4_ nanoparticles [58]—as no decrease in emission intensity was detected under intense (>550 kW cm^−2^) and prolonged (120 s) illumination with a focused 1064 nm laser (Figure S9). Moreover, acceptable PA properties (Figure S8) and minor intensity variation were observed throughout the ABS (Figure S10), which can be attributed to small local inhomogeneities in Tm^3+^ concentration and Y/F lattice composition (Figure S7 and Table S3). An enhanced PA signal was consistently observed at the edges and corners of the ABS, which we attribute to enhanced, excitation or emission light scattering. No additional optical effects like multiple reflections or light diffraction were found in our Tm^3+^ doped LiYF_4_ ABS, but some of these phenomena were discovered in Tm^3+^ doped β‐NaYF_4_ ABS crystals (Figure S11). In the latter crystals, we observed laser beam diffraction or reflection from the flat edges of the fluoride microcrystals surrounded by glass or air, which owes to the fact the excitation wavelength and beam size is comparable or smaller than the ABS dimensions. Such effects indicate the importance of consideration of these phenomena for future studies and developing optimized ABS substrates.

Beyond purely optical effects, the extreme nonlinearity of the photon‐avalanche process implies that PA luminescence can respond sensitively to perturbations arising from interaction between the exogenous ABS substrate and the overlaid sample [63], as well as to the intrinsic quality the ABS crystals themselves such as composition homogeneity, flatness, lack of cracks or photostability. A natural consequence of label‐free TAB imaging is therefore lack of intrinsic biochemical specificity (Chapter S1.3), i.e., the nano‐ micro‐structures are visualized because they are capable to attenuate the excitation beam. Nevertheless, in contrast to most label‐free super‐resolution approaches (Table S2 and Chapter S1.4), TAB microscopy offers an alternative form of functional specificity, originating from either wavelength‐selective attenuation of the laser photoexcitation beam by the sample (Figure S12) or from specific physical interactions—such as quenching or enhancement, between the sample and the exogenous ABS crystal [63]. Because optical attenuation is ubiquitous and only weakly specific compared with molecular recognition in fluorescent labeling or vibrational contrast in Raman microscopy, attenuation dominated TAB imaging alone would primarily provide enhanced morphological information. However, certain materials—including noble metal nanostructures, graphene, organic compounds or fluorophores, can, in addition to attenuating the excitation beam, interact more specifically with the ABS, through mechanisms ranging from distance‐dependent luminescence quenching to emission enhancement. To explore possible scenarios, we evaluated avalanching microcrystals (*Synthesis* *of* *3%* *Tm* *LiYF*_*4*_ *microcrystals* chapter) and employed differential rate equation model (*Modeling of PA properties* section) to theoretically describe, model and predict the susceptibility of the ABS luminescence to external and internal factors such as homogeneity of Tm^3+^ doping, excitation attenuation and non‐radiative quenching. Experimentally, such information can be readily achieved by performing local, pump power dependent (PPD) luminescence intensity measurements, which are technically affordable and can be seamlessly integrated into TAB imaging protocols. For most simple—pure attenuation case (Figure 3a), the excitation beam is attenuated without direct interaction between ABS and the sample. This results in apparent shift of the pump power dependent response toward higher PA thresholds (Figure 3a), while preserving the characteristic sigmoidal (“S”‐shaped) profile. However, owing to the high nonlinearity, derived quantities such as contrast enhancement (Equation 2, Figure 2f) and luminescence intensity ratio (LIR) (Equation 1, Figure 2e) exhibit non‐monotonic behavior as functions of the pump power (second and third rows of Figure 3). Maximum contrast improvement is obtained at pumping power set to a value near the transition between the avalanching regime changing to saturation regime (*I*_*P*0_ in Figures 1b and 3 vertical dashed line). Additional scenarios arise when non‐radiative energy transfer from the excited ABS to the sample leads to the quenching of either looping (Figure 3b) or emitting (Figure 3c) level. Such quenching effects are expected to occur at very short distances (<10 nm), and are particularly relevant for plasmonic or wide bandgap materials. The corresponding PPD profiles differ markedly from pure attenuation and from each other: quenching of the looping level not only reduces emission intensity but also alters the slope of the response as the quenching rate increases. Moreover, the attenuation induced LIR*(I*_P_) profile is more symmetric around central peak than the other two. It is straightforward to distinguish between two variants of quenching, because the quenching of the looping level not only reduce its intensity but also the slope in response to growing quenching rate. Nevertheless, the attenuation and looping level quenching profiles show many similarities, complicating unambiguous interpretation of the underlying interaction mechanism. To disentangle these effects, a control experiment can be performed, assuming sufficiently uniform PA properties across the ABS substrate. TAB imaging of the same specimen overlaid on a pristine ABS properties can be compared with measurements acquired using thin spacer layer—such as polymer film or an undoped ABS shell introduced between the sample and the exogenous ABS crystal. Whereas the former configuration captures the full complexity of attenuation and near‐field interactions, the latter suppresses non‐radiative coupling and isolates pure attenuation effects. Comparison of these two datasets enables separation of interaction‐driven contrast from attenuation‐only contributions, thereby extending TAB microscopy beyond purely morphological imaging toward interaction‐sensitive, label‐free contrast mechanisms.

**FIGURE 3:**
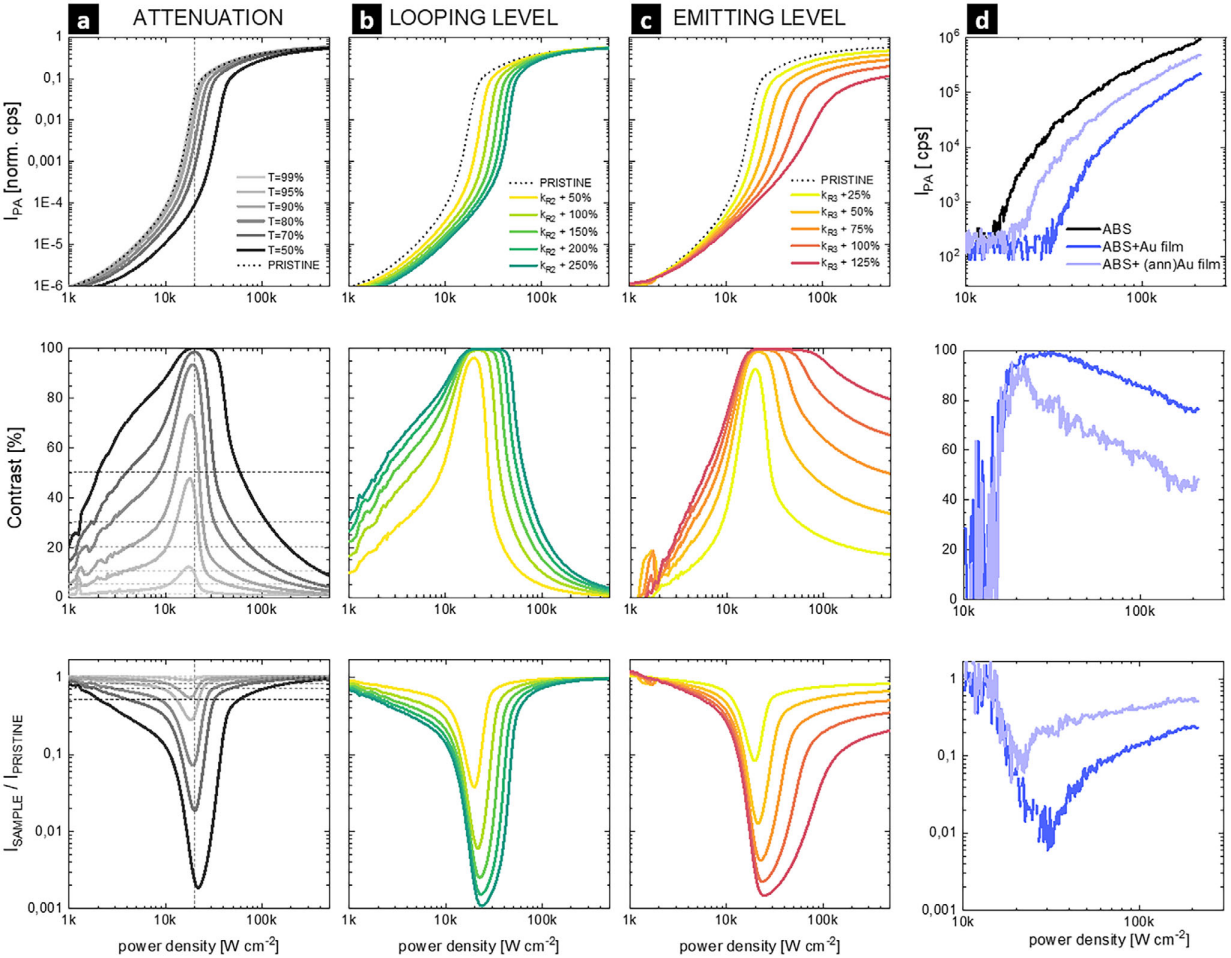
Simulations of pump power dependent PA emission intensity of the ABS in response to (a) pure attenuation of the excitation beam, (b) quenching of the looping level, (c) quenching of the emitting level—all plots were confronted with the pristine PA behavior (dotted line on panels a–c). The variability is presented in response to percentage change of: (a) transmittance (*T* = 99, 95, 90, 80, 70, 50%) of the sample; (b) radiative rates of the looping level (+50, 100, 150, 200, 250% of k_R2_) and (c) radiative rates of the emitting levels (+25%, 50, 70, 100, 125% of k_R3_) for 3% *T*m doping versus the pristine conditions and rates. The corresponding contrast (second row) and attenuation (*I*_S_ */I*_O_—third row) pump‐power dependent profiles are presented against profiles obtained for the pristine ABS. For pure attenuation horizontal dashed lines indicate intrinsic attenuation level. Panel (d) shows how the ABS reacts to pristine and annealed gold film (discussed later), placed in the path of the excitation beam in TAB configuration.

In addition to the effects discussed above, large‐area ABS crystals may exhibit compositional inhomogeneities that locally affect the CR efficiency and energy looping (Figure S8). Such variations directly influence the PA behavior and will can therefore affect both the interpretation and quantification of the observed TAB maps. Signatures of such behavior are apparent in experimental TAB maps (Figure S10a) and derived PA intensity statistics (Figure S10b,c) and PA properties (Figure S10d,e). Although SEM‐EDX maps of the ABS confirm an overall homogenous distribution of 3% Tm on the ABS surface, local variations of Y and F atoms distributions were detected, and thus validate our interpretation (Figure S7 and Table S3). Numerical modeling further indicates that modes changes in the cross‐relaxation rates (Δ*k*_CR_ = ‐10, ‐5, +5, +10%) can alter the PA behavior, leading to measurable variations in luminescence intensity ratio (LIR) (Figure S10d,e). These results underscore the importance of careful materials optimization and cautious interpretation of local TAB contrast variations. Nevertheless, in the present ABS crystals, the experimentally observed inhomogeneities extend over relatively large spatial regions rather than being sharply localized. As a result, they do not compromise imaging fidelity at the sub‐diffraction scale and allow reliable TAB imaging to be performed, providing a robust foundation for further experimental demonstrations.

Equipped with the fundamental knowledge on the ABS quality and homogeneity, and the precautions related to the interpretation of the PA signal, we performed TAB imaging of various objects. We began with thin gold film deposited directly on the glass microscopy slide (*Preparation of gold film* chapter, Figure S19). The ABS crystals were randomly dispersed on the glass slide with Au film structures. In Figure 4a, the ABS was laying on the edge of the thin Au film. While the white light contrast remained modest *C*_WL_ = 31% at 1064 nm, where the real PA interaction occurs, this contrast is equal to 40% (Figure S12). The loss of PA emission derived from TAB image (Figure 4b—black line, Figure 4c) exhibited markedly improved contrast of 90%, reflecting the nonlinear amplification of weak attenuation by the ABS. The cross‐section through the edge was studied versus pump power. A similar experiment on a homogenous Au film with a ≈3 µm thick scratch (Figure S23), also showed substantial contrast enhancement, from the original 21% to 96%. We next exploited the laser induced annealing (LIA) of thin gold film exposed to strong and tightly focused laser beam, aiming to demonstrate in situ modification and evaluation of this transformation. Macroscopically, upon LIA @ 1064 nm, gold nano‐micro structures were formed (Figure S20)—this was supported by the appearance of plasmonic peak at 550 nm (Figure S12f) and illustrated with SEM images (Figure S16). Because these metallic nanostructures do not feature optical activity corresponding neither to the excitation or emission bands of Tm^3+^ ions, as well as are expected to stay at distances beyond plasmonic interaction range, we expect no plasmonic interaction to be visualized in the course of TAB imaging. Nevertheless, subtle modifications of the pump‐power dependent S‐shape profiles could be noticed when comparing ABS on glass, pristine and annealed Au film (Figure 4e). This conclusion is further supported by the contrast and LIR profiles (Figures 3d and 4d), which resemble characteristic signature of emitting‐level quenching (Figure 4c) for the pristine gold film, which became reduced for the annealed version of the gold sample. Corresponding pump power dependent contrast C, PA intensity and width of the imaged edge is presented on Figure 4f. For the TAB experiments, a single ABS crystal was located at the edge of the fresh, thin Au film, and was imaged, both, in white light, as well as in TAB configuration (Figure 4g). Subsequently, localized laser annealing was performed in selected large area of the sample using raster scanning with purposefully high 1.02 MW cm^−2^ laser beam and imaged again in white light and in TAB configuration (Figure 4h,j). Laser annealing resulted in significant increase of transmission (Figure S12) at 1064 nm from 71.8% for pristine gold to 96.1% for the annealed region. Correspondingly, the TAB image revealed a pronounced recovery of the PA intensity in the area, where ABS crystal was shielded by the Au film, which clearly indicates the capability to perform in situ observation of the Au film modification (Figure 4j). Importantly, the ABS substrate exhibits exceptional photostability: no photobleaching, no photo blinking was observed, even under intense excitation. Consequently, the reliability of TAB imaging is limited primarily by technical factors such as long term laser stability and thermal stabilization of the PA substrate [64]. Although photodarkening has been reported in ANP, our experiments rule out this effect in bulk ABS, as no PA intensity degradation was detected under high pump power and prolonged 120 s exposure (Figure S9). Finally, the ABS substrates can be fabricated reproducibly using well‐established and scalable hydrothermal synthesis protocols, underscoring the practicality and robustness of TAB microscopy.

**FIGURE 4:**
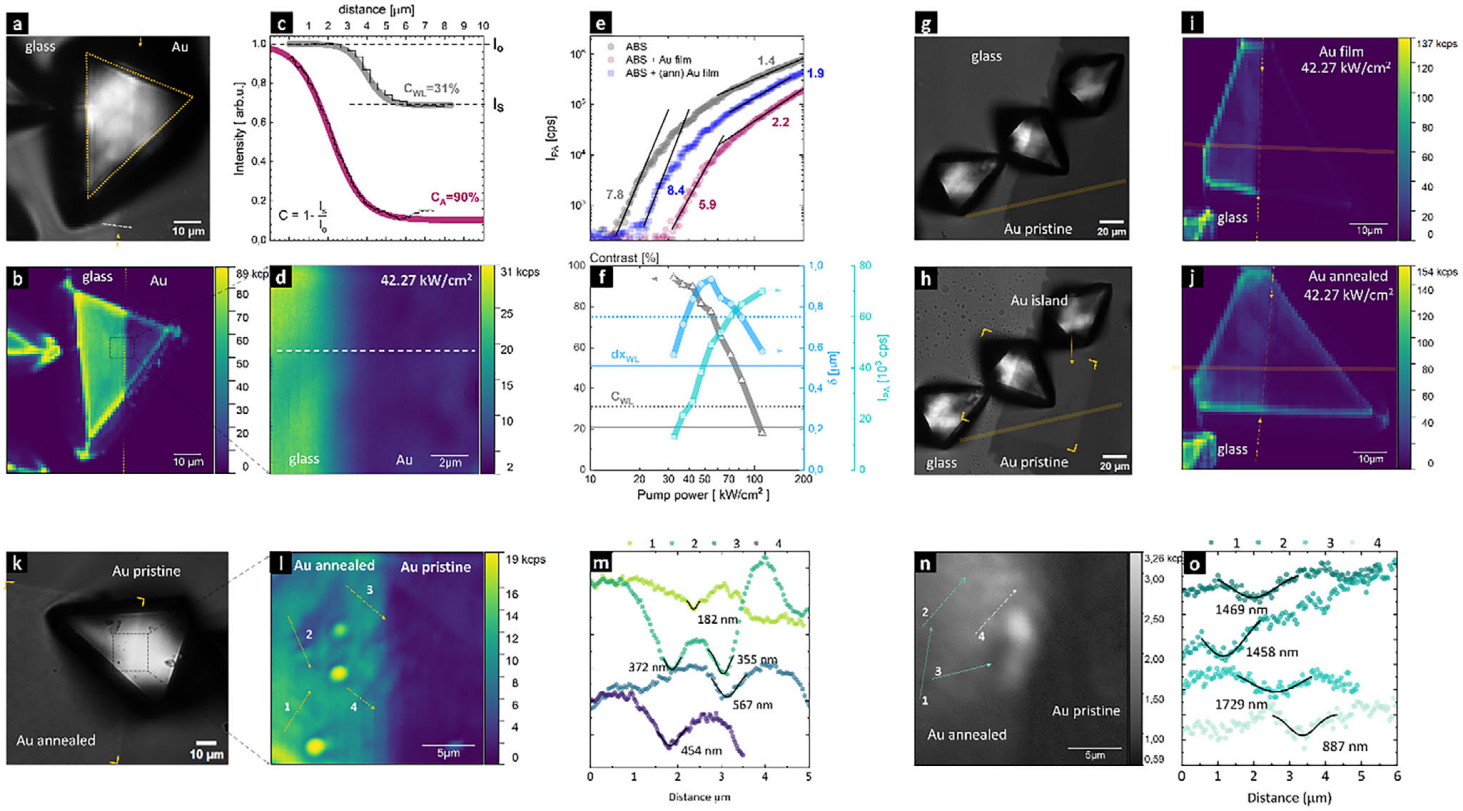
Representative self‐referenced TAB imaging of thin gold films and gold islands. (a) White light and (b,d) the corresponding TAB image of the ABS laying across Au film together with (c) cross section of intensity along the white dashed lines indicated on (a) and (d); (e) averaged pump power dependent PA emission intensity profiles for the bare ABS (black) and ABS through the fresh (purple) and annealed (blue) Au thin film and (f) corresponding (to (c)) pump power dependent contrast C (gray, left axis), intensity (green, right axis) and width (blue, right axis). (g–j) In situ monitoring of Au film annealing—(g) dynamic studies of Au film before (g–i) and after (h–j) laser annealing (the annealed region is indicated in (h), the cross‐sections through the lines indicated on white light (g, h) and TAB (i, j) panels are shown in Figure S4.11. (dx = 1 µm, dwell time 50 ms) (k) white light image of ABS laying on the edge between Au annealed and flat film and corresponding TAB (1064 nm excitation, detection 800 nm) (l) and back‐scattered (excitation and detection at 1064 nm) (n) images. The cross‐sections along the arrows indicated on (l) and (n) are presented on panels (m) and (o) correspondingly.

Thus far, we have primarily evaluated the contrast augmentation on macroscopic specimens, however, TAB imaging also enables enhanced optical resolution. To demonstrate this capability we performed local LIA of the Au Film and positioned the ABS‐es on such pretreated glass slide (Figure 4k; Figures S12–S15). This process of laser‐induced annealing (LIA) caused the smooth film to turn into separated gold nanostructures. White light (WL) images provides only weak indications of these LIA regions (Figure S15a–c), yielding images with insufficient contrast and limited structural details, even after software postprocessing such as histogram correction (Figure S15d,e). In stark contrast to WL, TAB images reveal many interesting structural features (Figure S15f,g) with substantially enhanced contrast. Quantitatively, the LIR increases to LIR≈150/90 = 1,6(6) for TAB imaging, compared with 1.09 for WL for raw, unprocessed images. Notably, the TAB image consistently reveal a characteristic doughnut shape morphology, which, based on SEM images (Figure S16), clearly confirms that this feature reflects the intrinsically non‐uniform nature of Gaussian‐beam‐induced annealing: in the center of the beam, the produced Au islands are larger and more sparsely distributed (case I), whereas the lower intensity laser beam wings produce Au islands that are smaller and more densely packed (case II). To explicitly demonstrate the enhanced optical resolution, we applied LIA over larger area of Au film using higher power stimulation, intentionally favoring the formation of case I islands, whose characteristic dimensions lie within the expected optical resolution range TAB imaging with *N* ≈ 10, as dictated by the nonlinearity in our ABS crystals. Indeed, SEM images confirmed the formation of Au islands with average FWHM size equal 84.7 nm, and average center‐to‐center spacing of 126.7 nm (Figure S16), well below 200 nm (Figure 4l,m). The pump‐power‐dependent TAB images further revealed enhanced optical resolution, namely, under stronger excitation power (closer to saturation region of PA performance) the TAB images are brighter but more blurred as compared to these captured with lower laser photoexcitation (at the most steep part of pump‐power curve), yielded fewer detected photons but substantially crisper in shape. In contrast, backscattered images (Figure 4n,o) failed to provide sufficient contrast to visualize the dense Au islands, revealing only weak differentiation contrast between island and homogenous Au film, accompanied by imaging artifacts. While majority of these Au islands are individual objects (Figures S20 and S21a—ROI_1,3_), occasionally during LIA process, some Au islands are forming larger, irregular structures (Figure S21a, ROI_2_). The smallest features sizes are equal to 182 nm (Figure 4m)—twice more than the sizes of Au islands and Au structures derived from the representative images (Figure S17), and definitely below theoretical diffraction limit of light (<450 nm for objective 100x NA = 1.45 at *λ* = 1064 nm), corresponding to an two‐ up to fourfold improvement in optical resolution. Importantly, pump power dependent optical resolution of the TAB images demonstrate the well‐known inverse dependence (*I*_P_)^−0.5^ (Figure S18).

As an all‐optical, technically affordable and cost‐effective approach that is not fundamentally constrained by the diffraction limit of light, the TAB imaging pave a way to support existing studies of biological or photonic sub‐100 nm objects with enhanced contrast and improved optical resolution. Nevertheless, as with any emerging new imaging technology, TAB requires a lot of further optimization in almost every aspect of the currently presented method. These include (i) the development of optimized ABS substrates—ideally multicolor, low‐threshold, high‐slope and high‐dynamic‐range PA materials fabricated as thin, flat, large‐area and compositionally homogeneous layers; (ii) refinement of imaging configurations (upright vs inverted geometries); and (iii) precise control of photoexcitation parameters. Selected challenges are discussed below, with a more detailed analysis provided in Sections S5 and S6. Frist of all, the TAB imaging operates in quasi far‐field—the virtual aperture is generated in far field, but the actual imaging mimics a near‐field imaging principle. Therefore, the increased distance between the sample or obstacle from the ABS crystal will hamper the optical resolution unless Bessel beams are employed. In addition, the TAB imaging does not intrinsically provide axial sectioning, and the laser beam for TAB raster scanning may potentially interact with the sample. These effects will strongly depend on the pumping wavelength and pump power density—the kilowatts per square centimeter used currently are a value acceptable for many applications, however it also remains sufficient to potentially induce thermal, plasmonic, autofluorescence or optical trapping effects. For example, occasionally, the focused beam leaded to rotation and translation of our ABS crystals, therefore one of the important future challenges to be addressed is the development of large and thin avalanching ABS substrates (Figure S26). Several routes may be pursued toward this goal, including: (i) synthesis of PA‐active nanosheet crystals [65], (ii) depositing avalanching thin shell on the non‐luminescent (micro‐bulk) crystals, (iii) self‐assembly of avalanching nanocrystals into flat, dense and highly uniform monolayer of ANPs [66], or (iv) ion implantation into to surface bulk single crystal. Achieving optimal TAB performance in terms of resolution and contrast further requires ABS substrates with exceptional compositional homogeneity, free from dopant clustering, structural defects or cracks, to ensure uniform backlighting and to avoid local scattering‐induced brightness variations. Moreover, development of various PA crystals operating across a broad range of excitation wavelengths—from UV, through Vis to the NIR regions—would significantly extend the palette of the obstacles available for imaging, providing additional insight into sample morphology, optical properties and spectral signatures beyond purely structural contrast.

At present, photon‐avalanche emission at 475 and 800 nm under 1064 nm excitation has been observed in Tm^3+^ doped microcrystals. In addition, emissions at 484, 525, 609, 642 nm (from Pr^3+^), 450, 475, 650 and 800 nm (from Tm^3+^) and 977 nm (from Yb^3+^) have been demonstrated under 852 nm photoexcitation. Numerous other wavelength combinations capable of supporting photon‐avalanche behavior have been reported (Figure S25) [54, 67, 68] and continuous progress in this area is anticipated [52]. This spectral flexibility introduces both opportunities and new challenges. On one hand, shorter wavelengths assure smaller diffraction limited spot, leading to smaller VA and, consequently, higher attainable optical resolution. On the other hand, shorter wavelengths are more likely to induce autofluorescence of the samples, which may increase background signal. Moreover, when both, emitting and looping levels fall into the excitation bands of possible endogenous fluorophores, resonant energy transfer or plasmonic interaction between the exogenous ABS and the sample may occur. Such interaction are strongly distance dependent and may either enrich or complicate the TAB contrast mechanism, depending on the application. At present, owing to the pyramidal, multi‐facet shape of the ABS crystals used in this study, TAB imaging has been implemented exclusively in an inverted microscope configuration, with the ABS placed directly on top of the sample. The up‐right microscope geometry would offer several important advantages. In such a configuration, the sample could be immobilized on a flat ABS surface and imaged from above, facilitating studies of thicker biological specimens, extended biomolecular assemblies, photonic devices and nanomaterials with non‐negligible thickness. The development of flat, planar ABS substrates is therefore expected to further expand the applicability and versatility of TAB microscopy.

Finally, the TAB imaging seems well suited for imaging of biological samples. As a proof‐of‐concept, white light (Figure 5a), TAB image (Figure 5b,c) and the corresponding, derived internal structure (Figure 5d) of a single eukaryotic Saccharomyces cerevisiae yeast cell demonstrates the ability to infer the spatial localization of intracellular organelles without the use of any fluorescent labels. The organelle assignment was based on their relative size and expected transparency [69]. In TAB image, the contrast is intrinsically negative, meaning the brighter the local PA intensity, the more transparent the sample is, therefore, structures such as nucleolus, endoplasmic reticulum or Golgi apparatus, which exhibit higher condensation of structures capable to attenuate the light passing through towards the exogenous ABS crystal, make the PA emission weaker than for the larger and water rich structures such as nucleus, lipid granule or vacuole. As with most label‐free techniques, the TAB imaging does not inherently provide optical sectioning along the axial direction, thus multiple absorption or scattering event at the optical path of the beam will accumulate. This constrains the TAB technique to imaging of very thin samples, unless tomographic like reconstruction strategies are employed. One may also note inhomogeneities on the TAB image beyond the cell (Figure 5b), which originates from the debris during yeast cell culturing and deposition on the glass slide, likewise the fact the foot of the exogenous ABS covering the few µm tall cell is tilted versus the optical axis of the excitation beam. These observations further emphasize the need for continued optimization of ABS geometry, compositional uniformity and optical configuration, particularly toward the realization of upright microscope implementations using flat, thin and planar ABS substrates (Figure S26).

**FIGURE 5:**
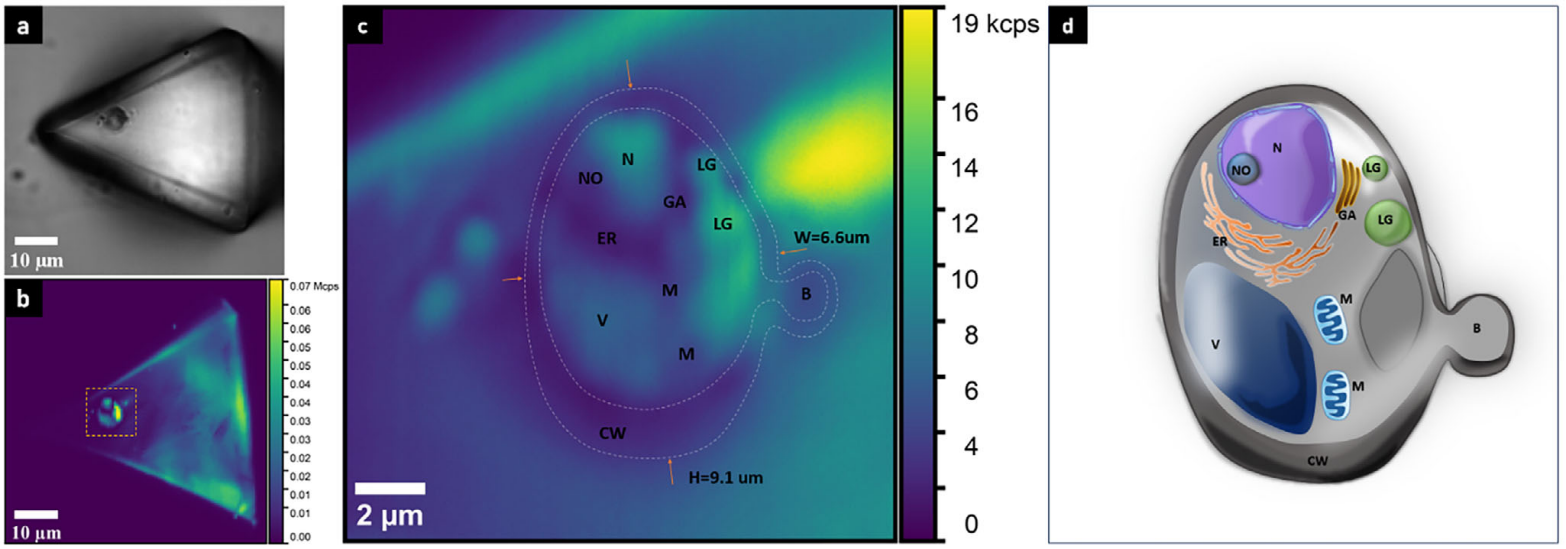
TAB imaging of yeast cell (Saccharomyces cerevisiae). a) White light image of a yeast cell covered with ABS crystal; b) low resolution TAB preview (*I*_P0_ = 117.54 kW cm^−2^, d*x* = 500 nm, d*t* = 20 ms), the cell marked with rectangle was imaged with higher resolution; c) high resolution TAB image (*I*_P0_ = 117.54 kW cm^−2^, d*x* = 50 nm, d*t* = 5 ms) and d) corresponding schematic outlook of the cell containing various organelles: CW—cell wall; LG—lipid granule; N—nucleus; NO—nucleolus; V—vacuole; M—mitochondrion; B—Bud; ER—endoplasmic reticulum; GA—Golgi apparatus.

Despite these anticipated challenges, TAB microscopy offers several compelling advantages. First, it is an all‐optical, non‐destructive, far‐field technique that enables to image pristine samples with no pre‐treatment, which are neither inherently fluorescent nor externally labeled. Owing to exceptional photostability of PA crystals and lack of blinking, negligible heat deposition and minimal thermal effects (Figure S24), photoexcitation wavelength that are incompatible with the endogenous chromophores and thus avoid radicals generation, as well as owing to low chemical and photo‐toxicity of this material (see Chapter S5), TAB microscopy shall permit prolonged observation times suitable for in situ time‐laps experiments. Although the PA threshold reaches 10–100s of kW cm^−2^ and exhibit prolonged risetimes (up to 10–100 ms), PASSI superresolution imaging with ANPs has been demonstrated with dwell times below 500 µs, which help to prevent exposing the delicate samples to excessive light exposure. TAB microscopy shall be particularly well suited for imaging of highly transparent biological samples. For example, the 1064 nm excitation and 800 nm emission [53, 56] used here, lies within a biologically safe spectral range [70] and does not induce detectable background autofluorescence of the sample. The only fundamental requirement imposed on the specimen is the ability to absorb or scatter the excitation wavelength used to trigger and modulate PA emission in ABS crystals. To further broaden applicability, the development of additional excitation‐emission PA wavelength combinations could be advantageous. Although not strictly required, the use of specifically absorbing contrast agents could further augment the imaging specificity when desired. The interaction of the sample with the PA substrate in situ, such as resonant energy transfer, plasmonic coupling or quenching of superficial layers of PA substrate [47] may further enhance imaging contrast but are not prerequisite for TAB operation. Importantly, systematic analysis of pump‐power‐dependent (PPD) responses can help disentangle simple attenuation effects from more complex interaction‐driven mechanisms, enabling more informed interpretation of TAB images of highly transparent biological or photonic samples and thin films. Moreover, owing to the intrinsic sensitivity of the photon‐avalanche process to temperature, mechanical strain and environmental conditions, TAB microscopy may be extended to visualize temperature or strain distributions and to probe bio‐interactions at the nanometer scale [60, 61, 62, 63, 64]. Finally, the large (anti‐)Stokes shifts and NIR‐Vis operation of TAB microscopy minimize sample autofluorescence, and make the technique inherently compatible with conventional fluorescence methods (wide field, confocal or super‐resolution) or near field techniques (SNOM, TERS, TENOM).

In conclusion, we proposed, devised and validated transmission avalanche backlight (TAB) microscopy—a fundamentally new imaging concept that enables label‐free visualization with enhanced imaging contrast and sub‐diffraction optical resolution. TAB microscopy not only reveals macroscopic features, such as contrast enhancement in thin Au films, but also enables investigation of microscopic phenomena, including the in situ formation of Au nano‐islands under focused laser irradiation. This approach allowed to visualize 36–130 nm (87 nm on average) large Au islands as <200 nm objects below diffraction limit of light. Moreover, TAB revealed homogenous and inhomogeneous regions occurring from interaction of Gaussian laser beam with 10 nm thin metallic film. Additionally, TAB imaging of yeast cells revealed internal organelles within single cell without using exogenous fluorescent labels. Further development of TAB technique shall focus on optimizing the nonlinear avalanche backlight substrates, refining upright microscope architectures, and advancing data acquisition and interpretation strategies for label‐free imaging. Nevertheless, owing to its non‐destructive, label‐free nature and far‐field operation, TAB microscopy offers a promising route for enhanced in‐vitro imaging of native morphology and pristine subcellular architecture, revealing unperturbed dynamics and functioning of living cells, subcellular structures or photonic devices at nanoscale, without the risk of their irreversible damage or functional alteration associated with excessive or exogenous labeling.

## 2 Materials and Methods

### 2.1 Contrast Enhancement

When the excitation beam passes through the attenuating medium of transmission *T* [%], then the original pumping power (*I*_P_) becomes attenuated ($I_{P}^{\prime }$), i.e., $I_{P}^{\prime }=T\cdot I_{P}$. However, in PA regime, the luminescence intensity *I*_L_ is proportional to the power *N* of pumping intensity *I*_P_, where *N* is the non‐linearity parameter in the power law: *I*_L_ = (*I*_P_)^*N*^. Therefore, the PA luminescence intensity equals $I_{L}^{\prime }={\left(T\cdot I_{P}\right)}^{N}=T^{N}\;\cdot I_{L}$, i.e., for *T* < 1, it is lower by a factor of *T^N^* as compared to the original, nonaffected luminescence intensity *I*_L_ (Figure 1b,d–f). In consequence, any non‐zero attenuation of this pumping light beam will be translated to disproportionally lower photon avalanche signal. The luminescence intensity loss can be quantified by luminescence intensity ratio (LIR) between the PA luminescence of the beam attenuated by the sample, related to the PA emission intensity of the non‐attenuated beam equals

(1)

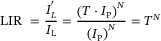

where the non‐linearity *N* is pump power dependent (*N*(*I*_P_)). To experimentally quantify the performance of TAB substrate, a contrast *C*(*I*_P_)—i.e., PA intensity loss parameter can be defined as

(2) $$C\;\left(I_{P}\right)=\frac{I_{L}-I_{L}^{\prime }}{I_{L}}=\left(1-T^{N\left(I_{P}\right)}\right)\;$$

and the absolute sensitivity

(3) $$S_{A}\;\left(I_{P}\right)=100\%\;\frac{\delta I_{L}}{\delta T}=100\%\;\frac{\frac{I_{L}-I_{L}^{\prime }}{I_{L}}}{1-T}=100\%\frac{1-T^{N\left(I_{P}\right)}}{1-T}$$

defines what relative change in PA emission intensity occurs in response to relative attenuation change. In other words, *C*(*I_P_*) measures how much the TAB augments the visualization of highly transparent subjects as compared to white light observation. For example, assuming photon avalanche nonlinearity of S = 20, the pristine attenuation by 1% (*T*  =  99%), will be evidenced by dimming PA luminescence from the incident one (*I_P_*)^*N*^ down to on (*T* · *I_P_*)^*N*^, which is equal to $I_{L}^{\prime }=T^{N}\;\cdot \;I_{L}={\left(0.99\right)}^{20}\;\cdot \;I_{L}=0.8179\cdot I_{L}$. This leads to translation of 1% change to luminescence intensity decrease by 18.2%, thus 8.2‐fold better contrast. Moreover, the transmission *T*(λ) is related to attenuation *A_T_* (λ) by a simple formula $T\left(\lambda \right)=10^{-A_{T}\left(\lambda \right)}$ and indirectly, through Beer‐Lambert Law *A_T_* [M^−1^cm^−1^]  =  ε*cl*, to the molar attenuation coefficient ε [cm^−1^], molar concentration *c* [M^−1^] of these species as well as the pathlength *l* [cm]. With an appropriate calibration, TAB imaging can therefore enable quantitative measurements of absorption, concentration or thickness of thin layers.

### 2.2 TAB Simulations

The Matlab 2019b simulations carried on Figure 2 assumed a diffraction limited excitation (λ_p_ = 1064 nm) gaussian beam is raster scanned through the phantom (gray layers or green particles) whose transmission *T*(*x*) is defined as gray area on Figure 2. The PA backlight substrate is therefore illuminated with a pump profile $I_{P}\;\left(x\right)=I_{PO}\;\cdot \exp\left(-\frac{{\left(x-x_{0}\right)}^{2}}{{\delta_{p0}}^{2}}\right)$, where *x*_0_ and δ_0_ are central position and full width half maximum ($\delta_{p0}=\frac{\lambda_{p}}{2\cdot NA}$) of the excitation spot respectively. This initial pump beam profile is multiplied by the transmission of the phantom *T*(*x*), thus the effective pump intensity profile is defined as $I_{P}^{\prime }\left(x\right)=T\left(x\right)\cdot I_{P}\left(x\right)$. For linear substrate the luminescence intensity is proportional to the pump intensity, but for photon avalanche case the luminescence scales with nonlinearity *N*. Moreover, every illuminated spot along the x axis generates a diffraction limited luminescence (λ_L_ = 800 nm) gaussian beam $\delta_{L0}=\frac{\lambda_{L}}{2\cdot NA}$, which after summing these contributions up $I_{L}\;\left(x_{0}\right)=\sum_{x}{\left[T\left(x\right)\cdot I_{P0}\cdot \exp\left(-\frac{{\left(x-x_{0}\right)}^{2}}{{\delta_{p0}}^{2}}\right)\right]}^{N}$ is ascribed as luminescence intensity at position *x*_0_. The signal is then normalized to the luminescence intensity achieved for the non‐perturbed case (for *T* (*x*) =  1). Similar simulations were performed in 2D, based on SEM image (Figure S21a,e), aiming to evaluate how the non‐linearity index *N* = 10, 20 and 30 (Figure S21b,c,d) affect the expected capability of TAB (Figure S21f) to resolve the features as small as 61 nm (Figure S21e).

### 2.3 Modeling of PA Properties

The TAB method is cable to not only enhance the contrast, but can also evidence some physical interactions between ABS substrate and the sample. When the PPD measured for the sample resembles that of pristine ABS (with the only difference the PA threshold being higher for the sample), the TAB image occurs exclusively due to attenuation or scattering of the sample and no physical interaction shall be expected. However, as soon as the PPD of the sample differs from the PDD of the pristine ABS, quenching of enhancement must be taken into account.

The modeling of PA behavior was based on the numerically solved differential rate equation presented previously, which were modified aiming to, on‐by‐one investigate the impact of attenuation (transmission *T = 1‐A* = 50, 70, 80, 90, 95 and 99 %), quenching of emitting (non‐radiative rate increase by *dk_2_* = 50, 100, 150, 200 and 250 % of the emitting level radiative rate) and looping (non‐radiative rate increase by *dk_3_* = 25, 50, 75, 100 and 125 % of the looping level radiative rate) levels (presented in Figure 3) and impact of cross relaxation (*dk_CR_* = ‐20, ‐10, ‐5, 5, 10, 20 % change versus the original CR rates). The impact of those factors was investigated by substituting *I*′_*P*_ =  (1 − *T*) · *I_P_* (for attenuation), $A_{3}^{\prime }=\left(1+\delta k_{3}\right)\cdot A_{3}$ (for emitting level quenching), $A_{2}^{\prime }=\left(1+\delta k_{2}\right)\cdot A_{2}$ (for looping level quenching) and *f_CR_* =  (1 + δ*k_CR_*) (for discussion on the homogeneity of doping across large PA crystals)

(4) $$\begin{array}{c} \frac{dn_{3}}{dt}=\frac{I_{P}}{h\nu }\;f_{ESA}\sigma_{ESA}n_{2}-\left(A_{3}+k_{3}\right)n_{3}-f_{CR}s_{31}n_{1}n_{3}+f_{CR}Q_{23}n_{2}^{2} \end{array}$$

(5) $$\begin{array}{cc} \frac{dn_{2}}{dt} & =\frac{I_{P}}{h\nu }\;\left(\sigma_{GSA}n_{1}-f_{ESA}\sigma_{ESA}n_{2}\right)-\left(A_{2}+k_{2}\right)n_{2}\\ & \;+\left(\beta_{32}A_{3}+k_{3}\right)n_{3}+2f_{s31}s_{31}n_{1}n_{3}-\left(f_{CR}Q_{22}+2f_{CR}Q_{23}\right)n_{2}^{2} \end{array}$$

(6) $$\frac{dn_{1}}{dt}=-\frac{dn_{2}}{dt}-\frac{dn_{3}}{dt}$$

where $\sum_{i=1.3}\;\frac{dn_{i}}{dt}=1$ and $\sum_{i=1.3}\;n_{i}=1$. The *I_P_*, *σ_GSA_*_,_ *σ_ESA_* parameters denote pump intensity, GSA and ESA cross section, *s*_31_ denotes the CR rate between level 3 and 1, *k*_3_ and *k*_2_ are non‐radiative multi‐phonon relaxation rates and *β*_ab_ denotes the emission branching ratio between state *n*_3_ and *n*_2_ */n*_1_ (*β*_32_ = 0.144; *β*_31_ = 1 – *β*_32_ = 0.856); *A_a_* is the radiative rate from level *a* (*a* = 2,3). Following ref [53] the other parameters are equal to *s*_31_ = *a*_cr_ · *c* ^2^ (*a*_cr_ = 160 s^−1^, *c* = 8 %), $Q_{22}=a_{\mathrm{uc}}\;\cdot \frac{c^{3}}{c^{2}+4.3^{2}}$ (*a*_uc_ = 9.00 s^−1^), $\;Q_{23}=a_{\mathrm{inv}}\;\cdot \frac{c^{3}}{c^{2}+4.3^{2}}$ (*a*_inv_ = 25.6 s^−1^). *A*_2_ = 162 s^−1^ and *A*_3_ = 636 s^−1^.

In the steady‐state conditions (i.e., when $\frac{dn_{i}}{dt}=0$ occurs for all levels), the population of the emitting level, *n_3_*, and thus, the relation between excitation and emission intensity (*A*_3_ *n*_3_) can be derived. These simulations are aiming to provide physical context and educative hints on the susceptibility of PA to the attenuation of the excitation beam, the non‐radiative depopulation of the emitting (*n*_3_) and looping (*n*_2_) levels, as well as the impact of the CR rates, rather than deliver exact solutions and fit our experimental data.

### 2.4 Reagents

Microcrystals were prepared using commercially available reagents. Yttrium oxide Y_2_O_3_ (99.999 %), thulium oxide Tm_2_O_3_ (99.995 %) were purchased from Chemicals101Corp, ammonium fluoride NH_4_F (≥98.0 %) was purchased from Sigma‐Aldrich. Ethanol (96 % pure p.a.) and nitric acid HNO_3_ (65 % pure) were purchased from POCH S.A. Ethylenediaminetetraacetic acid EDTA (≥99 %) and lithium fluoride LiF (99 %) were purchased from POL‐AURA.

### 2.5 Synthesis of 3%Tm^3+^ LiYF_4_ Microcrystals

LiYF_4_:Tm^3+^ microcrystals were synthesized by reported earlier hydrothermal synthesis method with some modifications [71]. Initially, the proper amount of yttrium and thulium oxides (9.7 and 0.3 mmol, respectively) were mixed with 50 mL of distilled water and ≈10 mL of nitric acid in a glass beaker. The beaker was then covered with a Petri dish and left on a hot plate set at a temperature of 140°C. Following the dissolution of the oxides, the beaker was left open on a hot plate for 16 h. Subsequently, 50 mL of distilled water was added to the dried precursor, and the mixture was evaporated to remove an excess of nitric acid. Subsequently, 1.06 g of EDTA and 30 mL of distilled water were added to the beaker. The mixture was vigorously stirred at room temperature for a period of one hour (solution 1). Simultaneously, 20 mmol of LiF, 60 mmol of NH_4_F and 30 mL of distilled water were vigorously stirred in a separate plastic container (solution 2). Subsequently, solution 1 was added dropwise to the solution 2 under vigorous stirring. The white, viscous mixture was then magnetically stirred for a duration of 30 min. Thereafter, it was transferred into a 100 mL hydrothermal synthesis autoclave reactor with PTFE‐lined vessel and placed in a laboratory drying oven. The temperature was set to 200°C, and the synthesis was carried out for 12 h before being cooled in an oven until it reached room temperature. The white precipitate was separated from supernatant by decantation and washed multiple times with distilled water and ethanol using an ultrasonic bath. The obtained microcrystals were dried on a hotplate at 100°C for 2 h. The structure and morphology of Avalanche Backlight Substrate (ABS) were presented on Figure S6.

### 2.6 SEM‐EDS Measurements for 3%Tm^3+^ LiYF_4_ Microcrystals

The morphology and chemical composition of Tm^3+^‐doped LiYF_4_ microcrystals were investigated by using a FE‐SEM FEI NovaNano SEM 230 (FEI Company as a part of Thermo Fisher Scientific) equipped with an energy dispersive X‐ray spectrometer EDAX Genesis XM4 with the resolution better than 135 eV and compatible with Genesis EDAX Microanalysis Software. Prior to the implementation of SEM imaging, the microcrystal sample was dispersed in alcohol and subsequently deposited onto a carbon stub in order to eliminate charging and drift problems. SEM images were recorded by using secondary electron (SE) at an accelerating voltage equal to 5.0 kV. The chemical composition analysis was conducted using the EDS technique for a randomly selected LiYF_4_:Tm^3+^ microcrystal. EDS measurements were acquired at an accelerating voltage of 30 kV using drift mode to enhance EDS maps quality. However, due to the inherent limitations of the EDS method in terms of the detection of light elements (*Z* ≤ 4), the EDS map of the Li element was not included and discussed in the paper.

### 2.7 Preparation of Gold Film

The substrate was cleaned by sonicating it in acetone, Isopropyl Alcohol (IPA), and water for 5 min. The substrate was then blow‐dried using N_2_ gas. A dehydration bake was performed at 120°C for 15 min. A patterned Au film with a thickness of 5 nm was deposited on the cleaned substrate using a thermal evaporator via the shadow masking technique at a vacuum of 7×10^−6^ torr.

### 2.8 SEM‐EDS Measurements for Au Films

The morphology of Au films was investigated by using the same equipment as for the Tm^3+^‐doped LiYF_4_ microcrystals i.e. FE‐SEM FEI NovaNano SEM 230 equipped with an energy dispersive X‐ray spectrometer EDAX Genesis XM4. Initially, the samples were positioned on the aluminum stub to rectify issues pertaining to charging effect. Subsequently, the samples were placed under the microscope to record SEM images at an accelerating voltage of 2.0 kV in a beam deceleration mode, with the objective of obtaining more detailed features of the samples.

### 2.9 Photophysical PA Properties Evaluation and TAB Imaging

Spectroscopic characterization of the samples was carried out using a home‐built fluorescence microscope based on the body of Nikon Ti‐U Eclipse inverted microscope and shown schematically in Figure S27. Excitation light at 1064 nm was provided by a single mode laser module (1064CHP, TEM_00_ Gaussian mode, 3SP Technologies), and controlled and thermally stabilized by a dedicated controller (ITC4001 controller + LM14S2 butterfly mount, Thorlabs). In order to automatically and precisely regulate the power of produced beam, motorized system of neutral density filters (NDF) was utilized. First of them (NDF1) was a gradient neutral density filter (NDC‐100C‐4 M, Thorlabs) used for gradual tuning of output power, while the other (NDF2) set of neutral density filters (FW121CNEB with preloaded ND filters, Thorlabs) was used for rough selection of the range of accessible light powers. After passing this module the beam was directed to the glass slide reflecting part of radiation to the optical power meter sensor (S121C+PM100, Thorlabs) and reference power value is monitored, which can be further used to determine the power density of the light illuminating the sample. Most of light (90%) is transmitted through the beam splitter. To overfill the back aperture of the objective, the laser beam is expanded using manual beam expander. Subsequently, the beam is directed by a dichroic mirror (DM2, DMSP900R, Thorlabs) to the lens (L) and enters the microscope's side port. Inside the microscope, light is reflected by the mirror (M) and, after passing through the lenses (L), enters the objective lens (O, Plan Apo λ 40x, NA = 0.95, Nikon, and oil immersion Plan Apo λ 100x, NA = 1.45, Nikon), where it is focused on the sample. The sample stage is integrated with an xyz piezoelectric stage (PI‐545 system, and E‐727.3CDA controller, Physik Instrumente) for raster scanning. The same objective lens is utilized for collecting of the sample emission and directing it back through the system of mirrors (M) and Lenses (L). Next, the beam is transmitted by the DM2 and passes through a set of filter wheels consisting of neutral density filters (ND3 = OD1.0, OD2.0, or OD4.0, Thorlabs), which are utilized to limit the intensity of signals exceed the dynamic range of the detectors. Once formed and pre‐filtered, the emission signal can be directed through two equal focal length bio‐convex lenses and a 200 µm pinhole (P200K, Thorlabs), which is kept at the focal point. The signal then passes through a band‐pass filter (BPF, FB800‐40 Thorlabs) before entering the avalanche photodiode unit (APD Counts Laser Components). The photon counts are integrated with a PCIe‐6351 National Instruments PCI card, which are synchronized with piezo stage movements. Alternatively, emission light can be directed to the setup dedicated to emission spectra measurements, composed of Andor Shamrock 500i Czerny‐Turner type monochromator with 3 gratings turret (GT) and equipped with the Andor Newton 920P‐BEX2‐DD CCD camera with active area chip cooled down to ‐60°C to limit the detector dark current. White light images were acquired with Andor iXion 897 512×512 EMCCD camera. Backscattered images were obtained similarly to the TAB images, by applying the microscopic dichroic mirror (DM1, DMSP 605T, Thorlabs) and APD band‐pass filter (BPF, 1050/25, EOTECHSPECTRA) filters to transmit 1064 nm laser light. No image postprocessing was applied to any WL, TAB nor BS images.

## Author Contributions

**S. Karmegam**: Methodology, Validation, Visualization, Writing – review & editing, Investigation, Data curation. **M. Szalkowski**: Methodology, Writing – review & editing, Conceptualization. **M. Misiak** Methodology, Visualization, Writing – review & editing, Investigation, Data curation. **K. Prorok**: Data curation, Methodology, Visualization, Writing – review & editing, Investigation. **D. Szymański**: Writing – review & editing, Visualization, Investigation, Data curation. **A. Bednarkiewicz**: Conceptualization, Investigation, Funding acquisition, Writing – original draft, Writing – review & editing, Visualization, Methodology, Validation, Software, Formal analysis, Project administration, Data curation, Supervision, Resources.

## Conflicts of Interest

The authors declare no conflicts of interest.

## Supporting information




**Supporting File**: advs78676‐sup‐0001‐SuppMat.docx.

## Data Availability

The data that support the findings of this study are available from the corresponding author upon reasonable request.
