# Supplementary material for: Label‐Free Super‐Resolution Imaging Using Non‐Linear Exogenous Photon Avalanche Backlight
Source: Adv Sci (Weinh). 2026 Nov 9:e78676. Online ahead of print. doi: 10.1002/advs.78676 (PMC13748906; doi:10.1002/advs.78676)
Supplement: Supplementary file 1 [file ADVS-9999-e78676-s001.docx]

SUPPORTING INFORMATION

**Label-free super-resolution imaging using non-linear exogenous photon avalanche backlight**

S.Karmegam^1^, M.Szalkowski^2,3^, M.Misiak^1^, K.Prorok^1^, D. Szymański^1^, A.Bednarkiewicz^1*^

1. Institute of Low Temperature and Structure Research, Polish Academy of Sciences, ul. Okólna 2, Wroclaw 50-422, Poland

2. Nanophotonics Group, Institute of Physics, Faculty of Physics, Astronomy and Informatics, Nicolaus Copernicus University in Toruń, ul. Grudziądzka 5, 87-100 Toruń, Poland

3. Institute of Advanced Studies, Nicolaus Copernicus University in Toruń, Poland

*E-mail: [a.bednarkiewicz@intibs.pl](mailto:a.bednarkiewicz@intibs.pl);

Contents

[Shortcuts:	2](#_Toc238236902)

[S1. Comparison of various super-resolution techniques	3](#_Toc238236903)

[S1.1 Super-resolution imaging methods	3](#_Toc238236904)

[S1.2 Contrast in label-free (super-resolution) imaging	5](#_Toc238236905)

[S1.3. Lack of specificity of label-free imaging	11](#_Toc238236906)

[S1.4. A comparison of TAB to other label-free techniques	11](#_Toc238236907)

[S1.5. Superresolution imaging with highly non-linear photon avalanche luminescent labels	14](#_Toc238236908)

[S2. Theoretical model of the nonlinear behavior of the backlight materials under Gaussian beam excitation	17](#_Toc238236909)

[S3. Characterization of the Avalanche Backlight Substrate	25](#_Toc238236910)

[S3.1. Characterization of structure and morphology of the ABS	25](#_Toc238236911)

[S3.2 Characterization of avalanching properties of the Avalanche Backlight Substrate	26](#_Toc238236912)

[S4. Characterization of Au film	29](#_Toc238236913)

[S5. Further considerations and future challenges for TAB imaging technique	36](#_Toc238236914)

[S5.1. The impact of local heating, toxicity and phototoxicity	36](#_Toc238236915)

[S5.2 Various PA colors for TAB microscopy	38](#_Toc238236916)

[S5.3 Future directions in development and optimization of the Avalanche Backlight Substrate	39](#_Toc238236917)

[S5.4 Future directions in understanding the PA interaction with the sample	41](#_Toc238236918)

[S5.5 Future directions in optimal composition of the ABS crystals	42](#_Toc238236919)

[S5.6. General considerations related to the TAB imaging & interpretation	43](#_Toc238236920)

[S5.7. The impact of PA nonlinearity	44](#_Toc238236921)

[S6. Optical setup	45](#_Toc238236922)

[S6.1 Setup outlook	45](#_Toc238236923)

[S6.2. Modifications to confocal microscope required to adopt TAB imaging	46](#_Toc238236924)

[S6.3. The stability of the photoexcitation source	46](#_Toc238236925)

[S7. Supporting information references	49](#_Toc238236926)

# Shortcuts:

**Related to methods:**

**TAB** – transmission avalanche backlight microscopy

**iSCAT** – *Interferometric Scattering Microscopy*

**MinFLUX** - minimal fluorescence photon fluxes microscopy

**SNOM** – *Scanning Near-field Optical Microscopy*

**STED** – *Stimulated Emission Depletion Microscopy*

**STORM** – *Stochastic Optical Reconstruction Microscopy*

**PALM** – *Photoactivated Localization Microscopy*

**RS** – Raman Spectroscopy

**TERS** -Tip Enhanced Raman Spectroscopy / Microscopy

**VM** - Label-free vibrational microscopy

**SOL** - Superoscillatory lens imaging

**SIM** – Structured Illumination microscopy

**CARS** - Coherent Anti-Stokes Raman Scattering

**Related to materials:**

**NB** – nanobody

**NL** – nanolayer

**EM** – energy migration

**ETU** – Energy Transfer Upconversion

**CR** – cross-relaxation

**PA** photon avalanche

**ABS** - avalanche backlight substrate

**ANP** – Avalanche nanoparticle

**NP** - nanoparticle

# S1. Comparison of various super-resolution techniques

There are many perspectives new imaging modalities may be compared to existing techniques. For TAB imaging method, at least three such features: (i) optical resolution, (ii) contrast and (iii) unspecific label-free features must be discussed.

## S1.1 Super-resolution imaging methods

***Table S1 Schematic comparison of selected sub-diffraction imaging techniques*** *(a-b) SNOM - scanning near field optical microscopy – the specialized hollow tip is used to confine volume of signal acquisition (I) large field of view excitation and fluorescence collection through the hollow tip; (II) excitation delivery and emission collection through the same hollow tip; (c) TERS - tip enhanced Raman spectroscopy – the signal (metal tip enhanced fluorescence or Raman scattered spectra) origins from interaction of sharp Au tip with fluorophore or molecules; (d) iSCAT – interference scattered microscopy relays of increased light scattering of nanoparticles, and requires image processing and reference image subtraction to augment tiny (c.a. 1%) variation of illumination light interference; (e) STED – stimulated emission depletion microscopy – the fluorophores are excited with 2 overlapping beams – the Gaussian shape excitation beam to excite fluorophores and doughnut shape depletion beam to deplete those fluorescent molecules which do no reside in the exact centre of the overlapped beams; (f) MinFLUX - localization method in which a structured excitation minimum is scanned around a fluorophore, and the molecule’s position is inferred from minimal detected photon counts with nanometer precision g)TAB – transmission avalanche backlight microscopy – the non-labelled sample modifies excitation beam, whose intensity is in-situ converted by a avalanching substrate to augment contrast and the optical resolution. NF tip, FF – far field, FF substrate – the far field excitation is combined with near field imaging, where the sample lays on the avalanching substrate, and this is the substrate with is actually scanned, Surface – due to scanning with tip, only Surface of the sample can be visualized, Flat – the imaging flattens the volumetric objects into flat image (like X-ray image does). NL – nano-layer, NB – nanobody. LEGEND: the arrows directed towards the nanolabels (NL) indicate photoexcitation, the arrows directed oppositely, indicate the PL collection, the dotted elliptical shape schematically indicate Point Spread Function (PSF), the halos around the NL, schematically indicate the photoexcited object, aiming to indicate which of the two NLs is actively detected. Scale:* high (◼◼◼), medium (◼◼), low (◼);

|  |  | | | | | | |
| --- | --- | --- | --- | --- | --- | --- | --- |
| **PARAMETER** | **SNOM (I)** | **SNOM (II)** | **TERS** | **iSCAT** | **STED** | **MinFLUX** | **TAB** |
| LABEL FREE | NO | NO | YES(Raman)/NO(PL) | YES | NO | **NO** | YES |
| EXCITATION LEVEL | 10-100 W/cm^2^, aperture size dependent | 10-100 W/cm^2^, aperture size dependent | 0.1-1 mW (10-100 kW/cm^2^ due to tip enhancement) | 10-100 W/cm^2^ | 0.1-1 kW/cm^2^ (excitation)  MW-GW/cm^2^ (depletion) | 5-50 W/cm^2^ | 50-500 kW/cm^2^ |
| OPTICAL RESOLUTION | ~50 nm | ~50 nm | ~5-10 nm | 200-300 nm | ~20-50 | <1 nm | ~50-100 nm |
| NEAR (NF) / FAR FIELD (FF) | NF tip | NF tip | NF tip | FF | FF | FF | FF substrate |
| 3D imaging / sectioning capabilities | Top surface | Top surface | Top surface | NO | YES | YES | Bottom surface |
| Speed of Imaging | ◼ | ◼ | ◼ | ◼◼◼ | ◼ | ◼◼◼ | ◼ |
| COMPLEXITY (setup/acq.) | ◼◼◼ | ◼◼◼ | ◼◼◼ | ◼ | ◼◼◼ | ◼◼◼ | ◼ |

## S1.2 Contrast in label-free (super-resolution) imaging

**Table S2. Comparison of various label-free super-resolution imaging methods.** LEGEND: Column 2 – DLim – diffraction limited resolution Column 3: FF-NF - far field (FF)/ near field (NF); Column 4: technical complexity high (◼◼◼), medium (◼◼), low (◼); Column 5: yes-◼ /no-🞏 /quasi true ≈◼ suitability for in-vitro, label-free and non-destructive imaging

| Method | Resolution  [nm] | FF/  NF | Technical complexity/ cost | Application  In-vitro/ label free/non-destructive | Advantages | Disadvantages | info | ref |
| --- | --- | --- | --- | --- | --- | --- | --- | --- |
| 1 | 2 | 3 | 4 | 5 | 6 | 7 | 8 | 9 |
| TEM | < 1 nm | FF | ◼◼◼ /  ◼◼◼ | 🞏/◼/🞏 | Sub nm resolution | Only thin samples, can be destructive for samples, high vacuum operation, expensive setup and operation | Imaging based on the detection of the electrons transmitted through the sample; allows for imaging of the details of the sample’s internal structure down to atomic level. Commonly combined with EDX accessory for elemental composition studies. | ^1^ |
| SEM | < 5 nm | FF | ◼◼◼/ ◼◼◼ | 🞏/◼/🞏 | <10 nm resolution | Only surface imaging, can be destructive for samples, expensive setup and operation | Imaging based on the detection of the back-scattered and secondary electrons; 3D surface reconstruction available. Commonly combined with EDX accessory for elemental composition studies. | ^2^ |
| AFM  Atomic Force Microscopy | < 1 nm | NF | ◼◼◼/  ◼◼◼ | ◼/◼/◼ | High resolution, down to single atoms, possibility of mechanical manipulation of the sample | Only surface imaging, requires dedicated tips, tip contamination, tip damage | Imaging based on the surface raster scanning with mechanical tip. Precise information about the topography of the sample | - ^3^ |
| 2Ph ABS  2 photon absorption | ~diff.limit | FF | ◼◼◼/  ◼◼◼ | ◼/◼/◼ | No exogenous labels | High laser powers required, fs operation | One of the essentia nonlinear optical methods in which a molecule simultaneously absorbs two low‑energy photons to reach an excited state, enabling characterization of its nonlinear optical properties under high‑intensity illumination.  Endogenous fluorophores are required. | ^4^ |
| STED + Raman | <50 nm | FF | ◼◼◼/  ◼◼◼ | ◼/◼/◼ | Resolution improvement, chemically specific mapping based on Raman signals | Complex, expensive setup | STED like Raman scattering - Stimulated Emission Depletion (STED) microscopy and Femtosecond Stimulated Raman Spectroscopy (FSRS). – complex – ps pump+fs probe+decoherence beam | ^5^ |
| Tip Enhanced  Raman Spectroscopy/  Microscopy (TERS) | Single nm | NF | ◼◼◼/  ◼◼ | ◼/◼/◼ | Indirect Composition mapping, reasonable signal | Requires tip scanning | Label‑free analytical method that combines a metallic nanoscale probe with Raman spectroscopy to amplify and confine the electromagnetic field at the probe apex, enabling vibrational (chemical) imaging with nanometer‑scale spatial resolution far beyond the diffraction limit. | ^6^ |
| Label-free vibrational microscopy (VM) | Diff.Lim | NF | ◼◼◼ | ◼/◼/◼ | Indirect Composition mapping | Weak signals | Label‑free imaging approach that uses molecular bond vibrations—detected via coherent Raman scattering or vibrational photothermal effects—to generate high‑sensitivity, high‑speed chemical‑contrast images of living cells and tissues | ^7^ |
| CARS Coherent Anti-Stokes Raman Scattering | Hundreds of nm, down to ~200 nm | FF | ◼◼◼/  ◼◼◼ | ◼/◼/◼ | Sensitive. Allows for selective imaging of the molecules based on their vibration energy; label-free operation | Requires three laser beams of wavelengths adjusted to sample. Only Raman-active molecules can be detected. | Raman spectroscopy enhanced due to resonant pumping. | ^8^ |
| PHOTOTHERMAL IMAGING | DLim (defined by probe beam) | FF | ◼◼◼/  ◼◼◼ | ◼/≈◼/≈◼ | Allows for detection down to the level of single molecules, possible label-free operation | Requires two coaxial beams and modulation, in forward detection mode two high NA objectives required.  Lack of resolution improvement. | Detection based on photothermally induced refractive index variations. | ^9^ |
| iSCAT | DLim | FF | ◼◼◼/  ◼ | ◼/◼/◼ | Allows for detection down to the level of single molecules, label-free operation. Relatively simple instrumentation. | Lack of resolution improvement. | Detection based on interference of the coherent light scattered on nanoscale objects. | ^10^ |
| Superoscillatory lenses (SOL) | No strict limit, λ/6 demonstrated | NF | ◼◼◼/  ◼◼ | ≈◼/◼/◼ | Large improvement of resolution, relatively easy implementation, no need for postprocessing algorithms | Required dedicated micropatterned mask | Scanning of sample with the light focused with dedicated mask. | ^11^ |
| transient absorption super-resolution microscopy (TAM) | ~100 nm | FF | ◼◼◼/  ◼◼ | ◼/◼/◼ | Fast, allows for super-resolution imaging of non-fluorescent samples | Technical complexity, high excitation powers, ultrafast laser pulses required | Imaging based on saturation of the electronic states with high-power donut-shaped beam, and probing the absorption in the central point of the beam; 3D sectioning avaliable | ^12,13^ |
| saturated coherent anti-Stokes Raman scattering (CARS) | 100-150 nm | FF | ◼◼◼/  ◼◼ | ◼/◼/◼ | Chemical specificity, improved lateral resolution without post-processing | High excitation powers, technical complexity | High excitation powers extension of CARS | ^14^ |
| Nanojet | λ/4 | ≈FF | ◼◼/  ◼ | ◼ /◼/◼ | Adaptable to regular confocal microscope setup, label-free, improved contrast | Specialized microlenses pattern required, limited field of view | Nano-jet formation with microlenses; 3D sectioning avaliable | ^15^ |
| OPRA (optical photon reassignment) confocal microscopy | λ/2 | FF | ◼◼/  ◼◼ | /≈◼/◼ | Resolution and contrast improvement, fast imaging, no post-processing required | Phototoxicity, photobleaching of the samples, imaging of deeper layers affected by scattering | Reflectance confocal microscopy with photon reassignment; 3D sectioning available | ^16^ |
| SIM – Structured Illumination Microscopy | DLim/2 | FF | ◼◼/  ◼ | ◼ /≈◼/◼ | Relatively easy to apply in wide-field microscopes, low-cost, fast | Requires collecting of several frames to reconstruct one image, requires image reconstruction algorithms | SIM and transillumination SIM | ^17^ |
| STED quenching microscopy | 30-50nm | FF  +  NF | ◼◼/  ◼◼ | 🞏/🞏/◼ | High resolution improvement available | 1.requires 3 excitation beams and spectral overlap + temporal synchronization  2. requires Ln3+ implantation in single crystal  3. specific for samples interacting (FRET) with the substrate | Super‑resolution fluorescence technique that uses a donut‑shaped depletion laser to switch off fluorophores around the excitation spot, leaving only a nanoscale central region emitting light and thereby achieving resolution far below the diffraction limit. | ^18^ |
| TAB | ~70-100nm | NF  +  FF | ◼/  ◼ | ◼/◼/◼ | 1. Currently T@1059 and T@852nm are available  2.photostable substrate  3.high photon counts (negative contrast) | 1.Disturbance to T@λ_P_ must be non-zero  2.no Z sectioning | Required: ABS substrate | This article |

## S1.3. Lack of specificity of label-free imaging

The TAB does not show explicitly (biological) specificity, as no external luminescent labels are incorporated into the sample. Of course, lack of such exogenous labels that are specifically prepared and directed towards specific structural or functional sites within the sample, may complicate the interpretation of the TAB images, like it does for other existing label-free methods (see Table S2).

Nevertheless, some specificity of TAB imaging may originate from a few features which are specific for the sample:

1) specificity of attenuation (i.e. absorption or scattering) by the sample, where the ABS excitation wavelength (here 1064 nm laser, but other wavelengths are possible too – Figure S5) is attenuated differently by different sample parts or compartments. For example:
A) Having a thin layer and knowing the absorption coefficient at this wavelength for bulk or more dense materials, one can derive layer thickness, number of layers, or inversely, knowing the thickness (together with properties of the ABS substrate), could enable measuring the absorption coefficient of these thin layers
B) While most of biological materials do not absorb light at 1064 nm, there are numerous other photon avalanche materials presented in Figure S5 (e.g. YbPr excitable at 852 nm and emitting on multiple lines in the visible around 480 and 620 nm). Thus, selecting appropriate ABS crystal, should enable imaging at specific wavelengths. Therefore, when the sample is rich in endogenous (e.g. NADH at 450 nm) or exogenous (e.g. PPIX at 630 nm) fluorophores capable to absorb the light at given wavelength, more specific imaging can be achieved.
C) The exogenous fluorescent molecules may still be used, either to be used for fluorescence microscopy or to augment the TAB contrast. Interestingly, while such approach neglects the concept of label-free detection, much lower labelling density should be feasible with less harm to the delicate samples.
2) specificity of physical interaction (e.g. quenching, energy transfer, plasmonic enhancement) between the ABS and the sample, which occurs at the energy corresponding to emission or looping energy levels within the ABS (see Figure 3, as well as reference ^19^ ). This interaction type can be investigated more accurately, by spot studying the full pump power dependence and analysis of the threshold, slope, intensity variation occurring in the sample as compared to the pristine ABS crystal. Most of these effects require very short, typically < 10 nm distances between the ABS and the sample.

Lack of biological specificity is posing a general issue of all label-free methods. But there exist some detection/ imaging methods, that have proven their suitability for biomedical studies without biological specificity. A good example is the iSCAT technique, which is capable to measure mass of biomolecules down to 10 kDa or visualize infection of eukaryotic cells without using specific labels.

## S1.4. A comparison of TAB to other label-free techniques

There are some common advantages and disadvantages of the label-free techniques, which originate mostly from non-specific imaging – i.e. the label-free techniques exploit some physical / chemical features of the samples that are visualised, which does not uniquely identify the observed object.

Out of existing label-free techniques, there are three very promising ones:

- **Structured Illumination Microscopy (SIM)** is a super-resolution fluorescence microscopy technique, which works by projecting patterned light onto the sample and computationally reconstructing high (typically 2-fold improved) resolution images using wide-field detectors (i.e. cameras). Because it relies on fluorescence contrast, it typically requires fluorescent labels or dyes to visualize specific structures. However, there are label-free adaptations of SIM, such as phase-contrast SIM or coherent SIM, which use intrinsic optical properties (like refractive index variations) instead of fluorescence. These are less common and usually applied in specialized contexts like live-cell imaging without staining. Biological samples (e.g., cells) are mostly transparent, so they don’t absorb light strongly. However, they alter the phase of transmitted light due to varying refractive indices. Phase contrast techniques (such as pristine Zernike phase-contrast or Nomarski method) combined with SIM offer numerous advantages. It allows imaging without fluorescent labels, which reduces sample preparation time and avoids chemical interference. It causes minimal phototoxicity and photobleaching. The technique improves resolution by about two times compared to conventional phase-contrast microscopy, and it preserves the natural state of the sample. It also works well for transparent specimens such as cells, tissues, and organoids. There are however some important disadvantages of Phase-Contrast SIM - the method provides lower contrast than fluorescence SIM because it relies on refractive index differences, which can be subtle. It lacks molecular specificity, so it cannot highlight individual organelles or proteins. The reconstruction process is computationally complex and requires precise calibration. Phase-based imaging is sensitive to optical aberrations and scattering. Finally, its resolution is still significantly lower than that of other super-resolution fluorescence techniques such as STED, PALM, or STORM. Taken together, the PC-SIM shall outperform TAB in terms of higher speed and lower phototoxicity. We however estimate that TAB may offer higher contrast for nanoobjects, higher and scalable optical resolution improvement ($\sqrt{N}$ >10 vs 2, for TAB and SIM, respectively) and quantitative imaging. Concerning technical aspects the two techniques are very different, i.e. SIM uses camera as detector and wide field patterned illumination while TAB is using single, fixed, semiconductor laser and piezo scanning, with a single photodetector. Both instruments should therefore be compatible and easily implementable into modern microscopes.
- The **Interferometric Scattering Microscopy** **(iSCAT)** is another label-free imaging technique that detects nanoscale objects by measuring the interference between light scattered by the particle and a reference beam. This approach provides extremely high sensitivity, allowing the visualization of single molecules, nanoparticles, and even viruses without the need for fluorescent labels. Because it does not rely on dyes, iSCAT preserves the native state of samples and avoids issues like photobleaching or phototoxicity. It also offers high temporal resolution, making it suitable for observing fast dynamic processes in real time, and can provide quantitative information about particle size and refractive index. However, iSCAT has some limitations. It lacks molecular specificity, meaning it cannot distinguish between different types of molecules without additional techniques. The method is highly sensitive to background noise caused by surface roughness or optical imperfections, which can reduce image contrast. Despite conceptual simplicity, the instrumentation is complex and requires precise alignment, and the signal strength depends on particle size and refractive index, making very small or low-contrast particles very challenging to detect. Additionally, iSCAT is best suited for thin samples or near-surface imaging and is less effective for thick specimens. The iSCAT outperforms TAB in imaging speed and setup complexity and cost. Although not yet available in the configuration comparable to iSCAT, the TAB imaging shall however offer similar advantages, and should similarly suffer from imaging of non-specific nanoobjects. Owing to non-linearity, TAB has a chance to further augment the contrast and optical resolution of imaging of small nanobodies and nanolayers below the limit of diffraction.
- The **super-oscillatory lenses** (**SOL**) uses advanced optical elements designed to generate light patterns that oscillate faster than the diffraction limit would normally allow. SOLs enable imaging at resolutions beyond the conventional diffraction limit without relying on fluorescent labels. In label-free applications, these lenses exploit the interaction of structured light with the sample’s intrinsic optical properties, such as refractive index variations, to reveal fine structural details. Because they do not require staining or tagging, super-oscillatory lenses also preserve the native state of biological specimens and minimize phototoxicity, making them suitable for live-cell imaging. The technique offers several advantages, including super-resolution capability in a simple optical setup and compatibility with transparent samples. It also avoids issues like photobleaching and chemical perturbation. However, there are notable limitations. Super-oscillatory lenses produce a very small high-resolution focal spot surrounded by strong side lobes, which can introduce artifacts and reduce image contrast. The method is highly sensitive to alignment and optical aberrations, and its performance can degrade in thick or scattering samples. Additionally, while it improves spatial resolution, it lacks molecular specificity and cannot selectively highlight particular structures without additional contrast mechanisms. Again, there are many common points between SOL and TAB, likewise they share similar issues natively related to lack of bio-specificity and thick, scattering samples. However, the concept of side-lobe free virtual aperture of the size below limit of diffraction ($\sim\frac{1}{\sqrt{N}}$) , simple ABS substrate (instead of complex SOL design) and direct compatibility with other types of microscopes (i.e. wide field fluorescence, laser scanning confocal, SIM, PC, STORM/PALM, STED, AFM, SNOM etc) shall be important advantages of the TAB technique.
- **Saturated coherent anti-Stokes Raman scattering** is a high-excitation-intensity extension of conventional CARS, operating in a regime where the vibrational Raman coherence becomes saturated due to depletion of the vibrational population difference. Under tightly focused illumination, the saturation intensity is exceeded predominantly at the centre of the excitation focus, leading to an intensity-dependent suppression of the resonant CARS signal. By selectively detecting the nonlinear, saturation-induced signal component, this effect enables spatial filtering and partial suppression of the non-resonant background. This mechanism is conceptually analogous to STED microscopy in that both rely on saturation-based suppression of signal generation to achieve sub-diffraction resolution; however, in saturated CARS the suppression arises intrinsically from the excitation intensity rather than from a spatially structured depletion beam. As a result, the optical instrumentation is simpler than in STED, since no donut-shaped beam is required. The technique employs pump and Stokes beams with high peak power densities (typically 10⁷–10⁹ W/cm²), coaxially focused onto the sample, with their frequency difference tuned to a specific molecular vibrational resonance. While this confers high chemical specificity in a label-free manner, it also limits versatility due to the need for precise spectral tuning and the use of high excitation intensities. In contrast, TAB imaging offers similar lateral resolution improvement (100-150 nm) and dwell times (tens of us) with single scanning beam of much lower intensity, with much higher versality (the request of specimen’s high transparency for the excitation beam is fulfilled by most of the thin organic films).

Imaging of highly transparent samples (e.g. thin layers, nanobodies, and hypothetically molecules, viruses, organelles, the interior of single layer of cells at the maximum) is beyond the scope of possibilities of most optical imaging label-free methods. Nevertheless, we believe TAB imaging can become a complementary approach to a few existing techniques, which neither show optical sectioning (AFM, iSCAT, SEM, SPR) nor biological specificity (AFM, iSCAT, TEM, SEM, SPR), and still, they have shown suitability to solve important questions in biology and nano-photonics.

**Sectioning**

Even though optical sectioning is currently impossible in TAB, one may speculate that oblique angle imaging developed for cryo-electron microscopy (cryoEM) and cryo-electron tomography (cryoET) ^20^ could be relatively easily adopted to TAB microscopy. While TAB cannot obviously compete with TEM resolution, the fact it uses photons which are harmless to delicate samples, augments the contrast without contrast labels and enable in-situ & in-vitro biological studies, makes it possibly interesting complementary technique to existing ones. As some of the label-free techniques have been developed for years or even decades and underwent intense development and optimization by numerous research groups, engineers and technical specialists, we are trying to look beyond currently, somewhat limited possibilities of TAB imaging. We strongly believe that development and optimization of the ABS thin layers and using up-right microscope will be the turning point. For example sharing similar issues related to inherent lack of specificity of imaging in thick samples, the concept of side-lobe free (as in SOL), virtual aperture of the size below limit of diffraction (~1/√S), augmented contrast & optical resolution, simple / cost effective ABS substrate, simple and cost effective TAB instrumentation and direct compatibility with other types of microscopes (i.e. wide field fluorescence, laser scanning confocal, SIM, PC, STORM/PALM, STED, AFM, SNOM etc) can potentially be important advantages of the TAB technique.

## S1.5. Superresolution imaging with highly non-linear photon avalanche luminescent labels

The use of photon avalanche (PA) for far-field sub-diffraction imaging was first proposed theoretically in 2019 by Bednarkiewicz et al., who showed that the extreme nonlinearity of PA emission versus excitation power density should, in a standard confocal raster-scanning microscope, narrow the effective point-spread function well below the diffraction limit — a concept later termed photon-avalanche single-beam super-resolution imaging (PASSI) ^21^. In the same year, an independent, complementary strategy was demonstrated experimentally by Denkova et al., who used bulk (non-avalanching) super-linear upconverting emitters to achieve 3D sub-diffraction imaging in a conventional confocal geometry, establishing that steep excitation–emission nonlinearity alone, without any additional depletion beam, can be harnessed for resolution enhancement ^22^. This approach was further developed by Plöschner et al., who combined super-linear excitation–emission behaviour with emission depletion in the same confocal setup, pushing the achievable sub-diffraction resolution of upconverting nanoparticle labels even further ^23^.

The first experimental realization of PA-driven super-resolution imaging in nanocrystals was reported by group of J.Schuck ^24^ with our contribution, demonstrating room-temperature photon avalanching in single Tm^3+^-doped nanoparticles with nonlinearity orders up to ~31, which translated into a lateral resolution better than 70 nm — about 7.5-fold finer than the diffraction limit of the 1064 nm excitation — obtained with a simple, inexpensive continuous-wave confocal setup and no computational reconstruction ^24^. This work confirmed the 2019 theoretical prediction and triggered rapid growth of the field. Liang et al. introduced migrating PA heterostructures with core–shell–shell architectures, reaching a 46^th^-order nonlinearity and multicolour sub-diffraction imaging of labelled cells down to 62–452 nm depending on emission channel ^25^, later extended to dual-colour 3D nanoscopy with a single low-power beam ^26^. Wang et al. demonstrated tandem PA energy transfer between the sensitizing and emitting sublattices, reaching a 41st-order nonlinearity applicable to a broader range of nanoscale emitters ^27^, while Wu et al. generalized this concept to cascade migrating PA networks spanning the full visible-NIR spectrum for multicolour super-resolution microscopy ^28^. Dong et al. exploited a dual-reservoir-level mechanism in Ho3+-doped nanoparticles to obtain parallel PA channels with tunable emission colour and 78 nm lateral resolution under single-wavelength excitation ^29^. Most recently, Liu et al. (2025) achieved an extreme nonlinearity exceeding 500 by sublattice reconstruction (Lu^3+^ substitution) in a single 27-nm avalanching nanoparticle, yielding 33 nm lateral and 80 nm axial resolution — the highest single-beam PA resolution reported to date ^30^.

Beyond raster-scanning PASSI, the steep and reversible photodarkening/photobrightening behaviour of avalanching nanoparticles far above threshold has been exploited for localization-based super-resolution: Lee et al. demonstrated indefinite, bidirectional near-infrared photoswitching of single avalanching nanoparticles, enabling indefinite photon-avalanche localization microscopy (INPALM) with sub-ångström localization precision ^31^. In parallel, PA nonlinearity has been combined with other optical-sectioning strategies to improve axial resolution: Liu et al. used self-interference field excitation together with a highly nonlinear avalanching response to reach isotropic sub-60-nm 3D resolution ^32^, and Pan et al. coupled ultrahigh PA nonlinearity with mirror-assisted bifocal 4Pi excitation to obtain a sidelobe-free axial resolution as fine as λ/33, without the instrumental complexity of conventional isoSTED ^33^. Collectively, these studies show that PA nanomaterials, unlike other nonlinear-optical or multiphoton super-resolution probes, allow diffraction-unlimited imaging with common continuous-wave lasers and unmodified confocal microscopes, while progressively pushing both lateral and axial resolution and expanding the accessible colour palette for multiplexed biological imaging.

# S2. Theoretical model of the nonlinear behavior of the backlight materials under Gaussian beam excitation

***General considerations***

*In the most general case, let’s consider the* ***3D Point Spread Function (PSF)*** *for a Gaussian beam. The approximation by the Gaussian function is reasonable and sufficient, because due to the non-linearity, the Airy rings will become negligible. The* ***PSF*** *is a fundamental concept in optics and imaging, which describes* ***how a single point source of light is imaged by an optical system****. Due to diffraction and imperfections, the image of a point is never a perfect point—it spreads out into a way dependent on the optical instrumentation and the wavelength of the light beam. The perfectly focused single mode laser beam is typically described and derived from the intensity distribution of a Gaussian beam in three dimensions, which includes both the transverse (x, y) and axial (z) dependence:*

$\text{PSF}(x,y,z)=\left( I_{0} \right)\cdot\left( \frac{\sigma_{0}}{\sigma(z)} \right)^{2}\cdot exp\left( -\frac{2\cdot(x^{2}+y^{2})}{{\sigma(z)}^{2}} \right)$	Eq.2.1

*where:*

- $I_{0}$ *is peak intensity at the focus,*
- $\sigma_{0}$ *is beam waist at the focal plane,*
- $\sigma(z)=\sigma_{0}\sqrt{1+\left( \frac{z}{z_{R}} \right)^{2}}$ *is beam radius at distance* $z$*,*
- $z_{R}=\frac{\pi\sigma_{0}^{2}}{\lambda}$ *is Rayleigh range,*
- $\lambda$ *is wavelength of the beam.*

*So, explicitly, the PSF describes the radial and axial intensity distributions of the excitation beam.*

$\text{PSF}(x,y,z)=I_{0}\cdot\frac{1}{1+\left( \frac{z}{z_{R}} \right)^{2}}\cdot exp\left( -\frac{2\cdot(x^{2}+y^{2})}{\sigma_{0}^{2}\cdot\left( 1+\left( \frac{z}{z_{R}} \right)^{2} \right)} \right)$	Eq.2.2

*Because the laser beam with such an PSF excites the photon avalanching material, in which the photoluminescence intensity distribution is related to the excitation intensity by a power law* $I_{PL}(x) = \left( I_{EXC}(x) \right)^{N}$*, so the* ${PSF}_{N}$ *of the photoluminescence equals*

$\mathrm{PSF}_{N}(x,y,z)=\left( I_{0} \right)^{N}\cdot\left( \frac{1}{1+\left( \frac{z}{z_{R}} \right)^{2}} \right)^{N}\cdot exp\left( -\frac{2(x^{2}+y^{2})}{\frac{\sigma_{0}^{2}}{N}\left( 1+\left( \frac{z}{z_{R}} \right)^{2} \right)} \right)$	Eq. 2.3

*Which confirms that due to the non-linearity (N>1), the axial term (along z direction of beam propagation) falls off faster with z because of the power N dependence, while the radial term (in x-y place perpendicular to optical axis) becomes narrower As the beam waist is defined as the length from the centre of the beam, at which the intensity falls to the value of* $I=I_{0} e^{-2}$ *, with N^th^ order nonlinear response the effective photoluminescence beam waist scales with* $1/\sqrt{N}$*.*

*Because in TAB imaging, we integrate the photoluminescence intensity within the* ${PSF}_{N}$ *volume, which is significantly smaller that the excitation beam itself, the TAB image will effectively be the result of convolution of this* ${PSF}_{N}$ *with the sample, thus the intensity variation will respond to smaller sample features and will assure improved spatial resolution.*

***Figure S1.*** *Comparison of PSF cross-sections at x-z and x-y planes of the gaussian 1064 nm beam for the linear case (N=1) and the PSF_N_ for the non-linear photon avalanching case (N=8). The original Rayleigh range* $\frac{\pi\sigma_{0}^{2}}{\lambda}$ *is reduced to* $\frac{\pi\sigma_{0}^{2}}{\lambda N}$*, while the original beam waist* $\frac{\lambda}{2NA}$ *is reduced to* $\frac{\lambda}{2NA\sqrt{N}}$*.*

***Simulations of TAB images***

*Similarly to the PASSI concept, which was first derived theoretically ^21^ and then evidenced experimentally ^24^, we have employed exactly the same, simple numerical simulation, which assumes non-linear response of PA substrate to the Gaussian beam. The algorithm is simple and has been added as additional information to the manuscript. The code is organized in the following way:*

1. *The Gaussian beam I_G_(x,y) scans over the photon avalanching backlight substrate (ABS) through the sample at spot S(x,y), where S(x,y) describes the transmission of the sample at position (x,y).*
2. *Therefore, each (x,y) region on the ABS experiences photostimulation with intensity of excitation I_G_(x,y)*S(x,y).*
3. *Because the photoluminescence I_PL_ is related to excitation intensity I_EXC_ with power law relation I_PL_ = I_EXC_^N^, where N is the non-linearity index, each of these ABS regions becomes fluorescent, generating photoluminescence with amplitude* $I_{PL}\left( x,y \right)=\left( I_{G}\left( x,y \right)\cdot S\left( x,y \right) \right)^{N}$*.*
4. *The luminescence originating from all these fluorescence spots is then integrated over the whole sample, which is assigned to the given spot position –* $I\left( x,y \right)=\sum_{x} \sum_{y} {{(I}_{G}\left( x,y \right)\cdot S\left( x,y \right))}^{N}$*. By performing raster scanning over the whole sample (x=1..X_max_ and y=1..Y_max_) one can easily generate the PASSI or TAB image.*

*The substantial difference between the PASSI (^21,24^) and the proposed TAB, comes from the fact that in PASSI, the substrate is non-fluorescent (S(x,y)=0) except position on the sample which contains avalanching nanoparticles (S(x_0_,y_0_)=1). In TAB imaging, the whole backlight substrate is fluorescent (S(x,y)=1), while any obstacles reduce the pump intensity passing through, which is due to scattering or absorption of excitation light on these object. This can be included in the analysis by simple assuming S(x,y)=T, where T is the transmission (T=1 fully transparent, T=0 fully opaque). More rigorous, analytical description of the improved resolution is provided below.*

***1D raster TAB scanning through the nano-obstacle***

*For sake of simplicity, let’s first consider a simple 1D Gaussian beam. In confocal microscopy, the excitation beam intensity profile is typically Gaussian*

$I_{EXC}(x)=e^{-2\frac{x^{2}}{\sigma_{0}^{2}}}$	*eq.2.4*

*For the non-linear luminescent material with non-linearity index N >1, the PL intensity scales as*

$I_{PL}(x) = \left( I_{EXC}(x) \right)^{N}$	*eq.2.5*

*Therefore, the PA emission intensity equals*

$P_{PL}(x)=e^{-2\frac{x^{2}}{(\sigma_{0}^{2}/N)}}$	*eq.2.6*

*Where the* ${2\sigma}_{0}$*is the excitation beam waist and* $x$ *is the distance from the beam centre. The use of nonlinear substrate effectively reduces the PSF of the imaging system by a factor of* $\sqrt{N}$*. In opposite to PASSI, TAB imaging assumes imaging of the homogenous PA substrate (i.e. avalanching backlight substrate – ABS), which is covered with nano-obstacles (e.g. nanoparticles of various kinds) or nano-layers.*

*A nano-obstacle (of transmission equal T) can be described by a window function, such that*

$f\left( x \right)=\left\{ \begin{matrix} 1 for \left| x \right|\geq W \\ T for \left| x \right|<W \end{matrix} \right.$	*eq.2.7*

*Where W is the half width of the window (i.e. 2W equals the size of the nano obstacle). For sake of simplicity, lets assume T=0%, which means a fully opaque nano-obstacle.*

*Now, imaging by raster scanning, means shifting the modified Gaussian beam (eq.6) through this window function (Eq.2.7), thus one may calculate convolution of both functions*

$g_{N}(x)=(I_{PL}*f)(x)=\int_{-\infty}^{\infty} e^{-2\frac{N\xi^{2}}{\sigma_{0}^{2}}}f(x-\xi)\text{ }d\xi$	*eq.2.8*

*The window function zeros out when* $\mid x-\xi\mid<w$*, i.e.,* $\xi\in(x-w,x+w)$*. So:*

$g_{N}(x)=\int_{-\infty}^{x-w} e^{-2\frac{N\xi^{2}}{\sigma_{0}^{2}}}d\xi+\int_{x+w}^{\infty} e^{-2\frac{N\xi^{2}}{\sigma_{0}^{2}}}d\xi$	*Eq.2.9*

*Next, the integrals may be calculated*

$\int e^{-\alpha\xi^{2}}d\xi=\frac{\sqrt{\pi}}{2\sqrt{\alpha}}erf(\sqrt{\alpha}\text{ }\xi)$	*Eq.2.10*

*where* $\alpha=\frac{2N}{\sigma_{0}^{2}}$*, so*

$g_{N}(x)=\frac{\sqrt{\pi}\sigma_{0}}{2\sqrt{2N}}[erf\left( \sqrt{\frac{2N}{\sigma_{0}^{2}}}(x-w) \right)+1-erf\left( \sqrt{\frac{2N}{\sigma_{0}^{2}}}(x+w) \right)]$	*Eq.2.11*

*finally*

$g_{N}(x)=\frac{\sqrt{\pi}\sigma_{0}}{2\sqrt{2N}}[1+erf\left( \frac{\sqrt{2N}(x-w)}{\sigma_{0}} \right)-erf\left( \frac{\sqrt{2N}(x+w)}{\sigma_{0}} \right)]$	*Eq.2.12*

*Where the erf(x)*

$erf(x)=\frac{2}{\sqrt{\pi}}\int_{0}^{x} e^{-t^{2}}\text{ }dt$	*Eq.2.13*

*indicates cumulative contribution of the Gaussian tails outside the window region. Now one can plot both, numerically (Eq.2.8) and analytically (Eq.2.12) calculated normalized convolutions to compare both solutions for various non-linearities (here N=7 and 30), as compared to the linear case (N=1). The nano-obstacle is defined by Eq.2.7, where we fixed W to 10% of the Gaussian beam size (*${W=0.1\sigma}_{0}$*). (****Figure S2****)*

***Figure S2.*** *Normalized, numerical (red) and analytical (blue) convolution plots of the Gaussian beam over the window function (grey).*

*One can also evaluate the N dependence of the full width half maximum (FWHM) of these local dip cross-section normalized to the nano-obstacle size (****Figure S3****).*

***Figure S3.*** *The full width half maximum (FWHM) of the cross section through the nano-obstacle versus the rising non-linearity N.*

*The results show, that for rising non-linearity of the ABS, the FWHM of the photon avalanche emission cross-section profile approaches the size of the nano-obstacle, which is this case was 1/10^th^ of the Gaussian beam size. Thus the sub-diffraction imaging can be anticipated. The results from numerical and analytical calculations are slightly different, which is likely due to discretization and convolution edge effects of the numerical calculations.*

*These calculations assume that the obstacle modifies only the excitation intensity and neglects any physical interaction (energy transfer, EM field concentration, quenching etc.) between the obstacle and the PA substrate. Such interactions can lead to very interesting physics as we have studied before ^19,34^. Namely further more extensive PA quenching or PA enhancement at the contact area between the sample and PA substrate can be anticipated. This requires a good control of the distance and more details studies – e.g. we have suggested that the PA luminescence properties (PL intensity, rise/decay times, spectra) of the given area should be evaluated versus the pumping power. Additionally, the performed calculations neglect optical effects such as light scattering, diffraction or interference.*

***1D raster TAB scanning through the edge***

*Now, similar calculations can be performed for a layer. Let’s define a the layer as an obstacle with a transmission equal T*

$f\left( x \right)=\left\{ \begin{matrix} 1 for x<0 \\ T for x\geq0 \end{matrix} \right.$	*Eq.2.14*

*Assuming* $T\in\left\langle0,1 \right\rangle$ *(*$T=0$ *for fully opaque layer and* $T=1$ *for fully transparent layer) and scanning the Gaussian beam through such obstacle, one can find a reasonably simple analytical solution.*

*Because we scan the ABS non-linear crystal with the Gaussian beam defined by Eq.2.2, we effectively scan the sample with the actually scan*

*The convolution* $g(x)$ *function as the Gaussian scans over the edge of layer* $f(x)$ *is given by:*

$g_{N}(x)=(I_{PL}*f)(x)=\int_{-\infty}^{\infty} I_{PL}(u)f(x-u)\text{ }du$	*Eq.2.15*

*Since* $f(x-u)=1$ *for* $x-u<0$ *(i.e.,* $u>x$*) and* $f(x-u)=T$ *for* $x-u\geq0$ *(i.e.,* $u\leq x$*), one gets:*

$$g_{N}(x)=T\int_{-\infty}^{x} I_{PL}(u)\text{ }du+\int_{x}^{\infty} I_{PL}(u)\text{ }du$$

*Eq.2.16*

*Expressing this in terms of Gaussian CDF(Cumulative Distribution Function)*

$\Phi(x)=\int_{-\infty}^{x} \frac{1}{\sqrt{2\pi}\sigma}e^{-\frac{u^{2}}{2\sigma^{2}}}\text{ }du$	*Eq.2.17*

*Makes the two integrals equal*

$\int_{-\infty}^{x} g(u)\text{ }du=\Phi\left( \frac{x}{\sigma/\sqrt{N}} \right) and \int_{x}^{\infty} g(u)\text{ }du=1-\Phi\left( \frac{x}{\sigma/\sqrt{N}} \right)$	*Eq.2.18*

*Thus*

$g_{N}(x)=T\Phi\left( \frac{x}{\sigma/\sqrt{N}} \right)+\left( 1-\Phi\left( \frac{x}{\sigma/\sqrt{N}} \right) \right)$	*Eq.2.19*

*and finally*

$g_{N}(x)=1+(T-1)\Phi\left( \frac{x}{\sigma_{0}/\sqrt{N}} \right)$	*Eq.2.20*

*where* $\Phi\left( z \right)=\frac{1}{2}\left[ 1+erf\left( \frac{z}{\sqrt{2}\sigma} \right) \right]$ *and* $\mathrm{erf} \left( z \right)=\frac{2}{\sqrt{\pi}}\int_{0}^{z} e^{-t^{2}}dt$

*This means, that the convolution is a smooth transition from 1 to* $T$ *governed by the Gaussian CDF, and the width of the transition through the sharp edge scales with* $1/\sqrt{N}$*. The analytically (Eq.2.20) and numerically (Eq.2.15) calculated PA intensity profiles give the same results (****Figure S4****).*

***Figure S4****. (a) the layer definition (grey) and non-linear dependent effective excitation beam for N=1,7, and 30. (b) Normalized, numerical (dashed line) and analytical (solid line) convolution plots of the Gaussian beam over the layer edge function (grey) for N=1,7, and 30. The transition width* $\Delta X=\left| x_{95\%}-x_{5\%} \right|$ *is defined as the distance between* $x_{S}=s\cdot I_{ABS}$ *positions, at which the s=95 and s=5% of the maximum PL intensity (*$I_{ABS}$ *at pristine ABS) is recorded.*

*This transition width* $\Delta X$ *through the edge, is represented in Gaussian width* $\sigma_{0}$ *units, and it depends on non-linearity index N in the way presented in* ***Figure S5****.*

***Figure S5.*** *The width of TAB cross section (measured as the distance along the x axis corresponding to transition from 5% to 95% attenuation from Figure S3) versus the non-linearity index N (1<N<100).*

This means, the sharp edge is imaged with a linear backlight substrate giving 160% of the ${2\sigma}_{0}$, which decreases below 20% of the ${2\sigma}_{0}$ (c.a. 8- fold resolution improvement) for high non-linearity index (N=100). This again supports the sub-diffraction optical resolution of raster imaging using non-linear ABS substrate.

The simulation code has been attached in the frame of open science.

# S3. Characterization of the Avalanche Backlight Substrate

## S3.1. Characterization of structure and morphology of the ABS

**Figure S6. Structure and morphology of LiYF_4_:3%Tm^3+^ ABS used in the experiments. a) XRD pattern of the obtained microcrystals and corresponding ICSD#73709 standard pattern for tetragonal LiYF_4_ (space group I41/a). b) Representative SEM images of LiYF_4_:3%Tm^3+^ microcrystals and c) schematic crystal structure of LiYF_4_.**

**Figure S7: Representative SEM image of 3% Tm^3+^-doped LiYF_4_ and corresponding elemental distribution for Y (green), Tm (yellow) and F (red).**

SEM image and EDS maps have been added SEM image and EDX elemental mapping of Avalanche Backlight Substrate surface shows uniform distribution of doping Tm^3+^ ions, but some inhomogeneous distribution of Y and F atoms.

**Table S3. Average atomic content of the chemical elements in Tm^3+^-doped LiYF_4_.** ^(*)^ The relative errors of EDS method are less than 2 %, 4 % and 50% for main (above 20 at. %), major (20 - 5. at. %) and trace (1 - 0.1) elements, respectively ^35^

|  | **No.** | **Chemical composition (at. %)^(*)^** | | | | |
| --- | --- | --- | --- | --- | --- | --- |
|  |  | **Li** | **Tm** | **Y** | **F** | **F/Y ratio** |
|  | 1 | - | 0.6 | 17.3 | 82.1 | 4.7 |
|  | 2 | - | 0.6 | 17.3 | 82.1 | 4.7 |
|  | 3 | - | 0.6 | 17.1 | 82.3 | 4.8 |
|  | 4 | - | 0.6 | 17.8 | 81.6 | 4.6 |
|  | 5 | - | 0.6 | 17.5 | 81.9 | 4.7 |
|  | 6 | - | 0.6 | 17.9 | 81.5 | 4.6 |
|  | 7 | - | 0.8 | 19.3 | 79.9 | 4.1 |
|  | 8 | - | 0.7 | 19.7 | 79.6 | 4.0 |

|  | **No.** | **Chemical composition (at. %)^(*)^** | | | | |
| --- | --- | --- | --- | --- | --- | --- |
|  |  | **Li** | **Tm** | **Y** | **F** | **F/Y ratio** |
|  | 1 | - | 0.6 | 17.3 | 82.1 | 4.7 |
|  | 2 | - | 0.6 | 17.3 | 82.1 | 4.7 |
|  | 3 | - | 0.6 | 17.1 | 82.3 | 4.8 |
|  | 4 | - | 0.6 | 17.8 | 81.6 | 4.6 |
|  | 5 | - | 0.6 | 17.5 | 81.9 | 4.7 |
|  | 6 | - | 0.6 | 17.9 | 81.5 | 4.6 |
|  | 7 | - | 0.8 | 19.3 | 79.9 | 4.1 |
|  | 8 | - | 0.7 | 19.7 | 79.6 | 4.0 |

## S3.2 Characterization of avalanching properties of the Avalanche Backlight Substrate

***Figure S8*** *Representative homogeneity of PA properties within single microcrystal. Using piezo stage positioning and laser spot (f=1.23 mm), five spots on the same crystal (a) were evaluated. Based on pump power dependence (b), maximum PA slope (c) and PA threshold (d) were derived. Each spot was measured 4 times and averaged.*

***Figure S9.*** *Confirmation of absence of ABS photodarkening. a) TAB map before beam exposure (dwell time dt= 10 ms,555.58 kW/cm^2^), b) WL image of Laser spot illumination, c) TAB map after laser exposure - 120 second with laser power density equal to 555.58 kW/cm^2^, dwell time dt=10 ms, d) zoom of area exposed to laser illumination (conventional setup).*

***Figure S10****. Impact of potential inhomogeneity of doping on the homogeneity of backlight in ABS crystals. (a) example of reduced PA intensities; (b) histogram of PA intensities (0..255 range of 8bit image) in the area marked on (a) by a dashed line, (c) PA intensity cross section along the dash-dot line; d) modelling of the impact of CR rates on the PA intensity, showing the comparison of pristine „S” shape (0%) versus cases where CR rates were decreased or increased (Δk_CR_ equal -20, -10, -5, 5, 10, 20% of the original k_CR0_); (e) correspondingly, the PA intensity modulation in response to k_CR_=k_CR0_+ Δk_CR_. The image (a) was acquired with a step size of 1 µm, a dwell time 10 ms, pump power 555.58 kW/cm^2^, and a non-confocal setup*

***Figure S11. Impact of potential optical diffractive effects on the performance of the backlight in ABS crystals. (a) Example of light diffraction within 7 µm large microcrystal on the PA emission intensities; (b) histogram of PA emission intensity (0..255 range of 8bit image) within the area marked by solid line on (a) and (c) PA intensity cross section profile along the dashed line on (a); (b) modelling of the impact of pump power on the PA intensity, showing the comparison of pristine „S” shape versus cases where local excitation intensity were decreased or increased (ΔI_P_ equal -10, -5, 5, 10 % of the original I_P_); (d) correspondingly, the PA intensity modulation in response to I’_P_=I_P_ + DI_P_ and (e) the corresponding relative change to the pristine (ΔI_P_=0) intensity.***

We speculate that PA intensity, being highly susceptible to energy looping and CR rates between neighbour Tm^3+^ dopants, is therefore highly susceptible to local variations of distances between neighbour ions or local crystallographic surrounding. Due to local segregation, either in response to diluting (leading to CR rate decrease) or concentrating (leading to CR rates increase) of Tm^3+^ dopants, or indirectly due to crystallographic sites variation may occur. Based on the SEM-EDX composition maps, our ABS crystals demonstrated homogenous distribution of Tm^3+^ ions, but slight variation of the Y and F atoms across the surface of the ABS crystal (Figure S2). Because TAB imaging is performed with a constant pump power, these local inhomogeneities of PA performance, may translate to local variations (up to 6-fold increase) of PA intensity. Further optimization of homogeneity of doping is required, or other approaches should be developed (tight self-assembly of homogenous nanocrystals, ions implantation into bulk substrate) to assure more homogenous ABS backlight intensity.

# S4. Characterization of Au film

***Figure S12. Macroscopic evaluation of Au film transmittance at 1064 nm of pristine and laser annealed Au film. Using the experimental setup similar to TAB imaging (d), the power of 1064 nm laser passing through the sample was measured directly to be a) on bare glass P_1064_ = 5.22 ± 0.03 mW, b) on pristine Au film P_1064_ = 3.75 ± 0.07 mW, c) on an annealed Au film P_1064_ = 5.02 ± 0.025 mW. This gives transmission of pristine Au film T_FAu_ =(3.75/5.22)*100 = 71.85 ± 1.75% and transmission of the annealed Au film T_AAu_ = (5.02/5.22)*100 = 96.14 ± 1.03%. The laser power for transmission measurement were below annealing threshold. The purposeful annealing (c) occurred in course of a raster scanning with high pump power P = 1092.78 kW/cm^2^ and dwell time dt= 10 ms. (e) transmittance / contrast spectra and (f) absorbance spectra of pristine and annealed gold film.***

***Figure S13. Laser annealing of Au film that was used in experiments on Fig.4. Laser annealing of pristine Au film at varying power density and exposure time, the power density was gradually increased from 82.9 kW/cm^2^ to 1420.5 kW/cm^2^, with each laser spot position shifted using piezo stage, a) dwell time dt = 50 ms, c) dwell time dt = 1000 ms; b), d), cross sections of the raw WL images along the lines indicated by colour arrow lines; e) integrated areas of the profiles presented on panels b) and d) versus laser power density, for both 50 ms and 1000 ms laser exposure times. The error bars indicate the standard deviation of the measured area.***

***Figure S14. Laser annealing of Au film. Pristine Au film (frame #0) was exposed to laser TAB scanning with power of a) 71.87 kW/cm^2^, b) 499.39 kW/cm^2^ and d) 1.02 M/cm^2^, and dwell time dt= 10 ms; c),e) cross sections of the WL images along the line indicated. The scanning was repeated up to 5 times (frames #1 .. #5). After annealing occurred, no sign of further gold film modification is observed.***

***Figure S15. White light (WL) and Transmission Avalanche Backlight (TAB) images after laser induced annealing (LIA). LIA was applied to different spots of the Au islands (1092.78 kW/cm^2^), and then the ABS crystal was placed on the spot. (b) is a zoom of white Region Of Interest (ROI) from (a), (c) is a zoom of white ROI from (b), (e) is presenting contrast corrected version of (c), while (d) shows schematically image histogram before and after contrast correction (ImageJ). A TAB map was generated for regions of interest (ROI) at varying photoexcitation pump powers. f) TAB image of ABS and g) Zoom region of ROI, h) TAB map of i) ROI 1 (step size 100 nm, dwell time 5 ms), and ii) ROI 2 (step size 100 nm, dwell time 30 ms) at various photoexcitation pump powers.***

***Figure S16. Analysis of LIA region. (a) TAB map of the LIA region of the ABS crystal (b,d,e), SEM image of the LIA of the Au islands at various magnifications. Distribution of (c) Au particle size and (f) particle distances in the center and the edge of LIA treatment.***

***Figure S17. The effect of lased induced annealing on the Au film. (a) The edge of the LIA region imaged by SEM. The ROI on (a) is magnified in (b), and the two ROIs of the LIA (red) and pristine (green) regions have been further magnified on (d) and (c) respectively.***

***Figure S18 (a) TAB images of the Au islands at rising photoexcitation pump powers, b) cross section of the TAB map, c) Full Width Half Maximum of the first peak position together with a logistic fit (grey) and allometric (black); the latter fit was done for 25-47 kW/cm^2^ and follows the expected I_P_^-0.5^ relationship. Subsequent imaging was leading to slight sample translation as the outcome of piezo-stage movement.***

***Figure S19****.* ***AFM scan of the Au film****, (a) a map and (b) cross section along the line present on (a). AFM Microscope was Nanosurf FlexAFM with C3000 Controller. Images were obtained in tapping mode and the cantilever type was HQ:NSC18/Al BS*

***Figure S20****. AFM images of Au islands created by LIA of Au films.*

***Figure S21.*** *Comparison of (a) experimental SEM image of Au Islands with (b,c,d) TAB simulated images for non-linearity index N=10,20 and 30, correspondingly. The SEM shows inhomogeneities of the Au islands – see region of interest (ROI) 1-3. (e) The cross-section of the SEM image (e) show features 36 to 132 nm large. The SEM image was used as a TAB phantom whose cross section along the line 3 is presented for SEM (f, top) for TAB imaging (f, bottom). For higher non-linearities, all, even smallest features present on SEM can be reproduced on TAB image simulations. The phantom was based on real SEM image, i.e. the 8-bit gray scale SEM image ISEM(x,y) was scaled to get the phantom image Iph(x,y), as Iph(x,y)= 1.0-(T/100)*(I_SEM_(x,y)/255) with modulation depth 20%. The 2D imaging was performed following the method described in TAB simulation chapter in major article.*

***Figure S22. In-situ monitoring of Au film laser annealing.*** *White light images (a,b) and TAB images (c,d) and PA emission intensity cross sections (e,f) of the ABS intensities before (a,c) and after (b,d) laser annealing. The annealing performed with 42.3 kW/cm^2^, TAB imaging – step size 1 um and 50 ms dwell time.*

***Figure S23. (a) White light and (b) TAB imaging of Au thin film with a scratch. (c) the cross section of raw white light image (contrast equals 26 and 21% for the scratch observed directly on bare film and through the ABS) and the film compared with the corresponding cross section (from (d)) of the TAB image (contrast equals 96%).***

# S5. Further considerations and future challenges for TAB imaging technique

Like any new technology, TAB requires a lot of further optimization in almost every aspect of the method, including (i) the ABS crystals (both multicolor, low threshold, high slopes, high dynamic range, ABS as thin, flat, large, homogenous layers) (ii) imaging configuration (upright s inverted). Some of these challenges have been discussed in the manuscript, some more in-depth discussion is presented in the following sub-chapters.

## S5.1. The impact of local heating, toxicity and phototoxicity

The 10-100 kW/cm2 pump intensities required for TAB imaging seem large, but this is still similar or less as compared to conventional confocal microscopy and 10-100 less than in depletion beams used in STED microscopy. Moreover, the 1064 nm wavelength used here is weakly absorbed by , for which most of biological samples are highly transparent, which means no excessive energy is deposited in the volume of the sample. For example, Artur Ashkin, the inventor of optical tweezers, has demonstrated back in 1989 year, yeast cell division under optical tweezers^36^ configuration with the 1064 nm focused laser. Actually, while the pump power density shall be acceptable and harmless for the biological samples, the optical forces may be more problematic because it may trap the nanobodies present in dense liquid environment. Nevertheless, the optical force drops rapidly for particle size getting smaller.

During raster scanned imaging with focused laser beam a heat deposited to the delicate samples should be considered. Based on out previous studies on the impact on temperature on PA emission, under excessive local heating we would expect rising PA threshold and hampering of TAB imaging ^37,38^, which was not the case. Moreover, the quantum yield of the photon avalanche has been estimated to be high ^24^, thus there is no large heat deposition in the ABS crystal expected. Moreover, such high pump power densities are delivered to diffraction limited spot size (ca. 1 um diameter), of the bulk-like crystal with good thermal conductivity and heat dissipation capability.

However, in the course of additional experiments, we noted a small but systematic rise of PA intensity by 7.5 and 14% for the Au film and Au islands after 80 s illumination (**Figure S24**).

***Figure S24. Evaluation of thermal, laser induced effects.*** *Long-term (80 seconds) kinetic studies of PA emission intensity in single spot, under 1064 nm excitation beam ( 34 kW/cm2, which is typical for most of our TAB images) for ABS crystal placed on (a) bare glass slide, (b) previously Au annealed region, (c) pristine Au film. The PA intensity and histograms are presented on (d). The +14% and +7.5% PA intensity growth for annealed and film Au samples was noticed around 80s after switching on (time t=0 s) the 1064 nm laser. This can be assigned to thermal effects resulting from interaction of laser NIR beam with gold sample. Typical TAB dwell time is << 100 ms, which is much shorter than the time required to induce any thermal drift.*

Concerning **phototoxicity** - this effect is by definition absent in the course of imaging with lanthanide doped nano/micro-materials. The optical transitions in lanthanide doped materials exploit f-f transitions, which are hidden behind 5s and 5p electrons, thus the 4f electrons does not take part in any types of chemical reaction. Moreover, lanthanide ions are embedded in the inorganic LiYF4 crystal (replacing Y3+ ions), thus radicals production is absent (in opposite to numerous organic fluorescent molecules). Thus, phototoxicity can be excluded.

The other question related to toxicity in general could be potentially related to chemical toxicity, but these LiYF4 materials, either in bulk or nanocrystalline form have demonstrated good biocompatibility ^39^, but again, the label-free TAB imaging does not require incorporation of any artificial, exogenous materials to living cells, thus the chemical toxicity issues can be also excluded from the risk analysis. The last question could be raised, when the laser invoke phototoxicity (radical oxygen species production) of the endogenous chromophores, but none is absorbing that wavelength and singlet ® triplet transition are firstly forbidden. Also the singlet state energy is 0.98 eV above ground state (this corresponds to 1270 nm), thus direct singled oxygen generation is absent.

## S5.2 Various PA colors for TAB microscopy

Because the principle behind TAB microscopy relays on the interaction of excitation beam with sample, one may need more effective wavelengths also in the visible spectral region ^34,40^. The attenuation of ABS excitation light may vary across sample compartments / compositions, enabling structural and compositional insights. For instance, knowing the absorption coefficient for bulk materials should allow for estimation of thin-layer thickness or, conversely, determination of absorption properties when thickness is known. While most biological materials do not absorb at 1064 nm, other photon avalanche systems (e.g., YbPr excitable at 852 nm) can be selected for imaging at specific wavelengths, improving contrast in samples rich in endogenous (e.g., NADH) or exogenous (e.g., PPIX) fluorophores. Additionally, exogenous fluorescent labels can augment TAB contrast with lower labelling density, minimizing sample damage. Beyond optical attenuation, specificity also arises from physical interactions—such as quenching, energy transfer, or plasmonic enhancement—between ABS and the sample at emission or looping energy levels. These effects, requiring sub-10 nm proximity, can be probed by analysing pump power dependence, thresholds, and intensity variations relative to pristine ABS crystals.

**Figure S25. Optical properties of major tissue components. I^st^, II^nd^ and III^rd^ optical transmission windows of tissue are shown. Excitation and emission wavelengths of known and new PA materials (black – possible PA excitation wavelengths, color – PA emission wavelengths) for various lanthanide PA ions.**

## S5.3 Future directions in development and optimization of the Avalanche Backlight Substrate

Although currently available inverted microscope configuration and currently available ABS crystals are suitable for proof-of-the-concept studies and demonstrations, they are limiting types and scope of samples suitable for TAB imaging. Although the LiYF_4_ microcrystals used here have shown satisfactory large size (triangle food 38.9 x 38.3 x 30.7 μm), but their irregular bipyramidal shape, angled facets resulting in multiple excitation laser reflections within the crystal makes the imaging volumetric samples like cells difficult at the moment. Moreover, the inverted microscope configuration means, the actual sample must be covered with these bipyramidal microcrystals. These difficulties should and can be relatively easily overcome. The advantage of the inverted configuration is the use of oil-immersion between the objective and bottom glass slide, which ultimately enables large numerical aperture observations.

Convenient imaging of biological samples would thus require an ideally infinitely large, flat and thin ABS substrates and imaging performed in the up-right microscopic configuration to direct the laser excitation beam through the sample (e.g. cell), down towards the ABS crystal (Figure S26d,e or f). To assure high numerical aperture observations, water immersion objectives would be of interest, and typical working distance of a few hundreds of micrometres would be sufficient to observe most thin bio-samples such as cell cultures.

**Figure S26. Various configurations of TAB imaging using inverted and up-right microscopes**. (a) LiYF_4_ micro-crystals with ca. 30x30 um large flat surface is used as ABS. Due the shape, only inverted fluorescence microscope can be used with the ABS covering the actual sample. This configuration has been used in our work; (b) to avoid volumetric PA emission, these large LiYF_4_ crystals should be developed by covering the undoped – non-luminescent LiYF_4_ core with avalanching thin shell. Such crystals are currently unavailable to our knowledge; (c) hexagonal NaYF4 large microcrystals (8 um x 1 um thick). The flat surface could enable imaging in inverted (c) and up-right (d) microscopes, however, we have tested such crystals and with the size similar to the wavelength of light, optical artifacts and significant scattering from the edges have been noticed thus we avoided these materials from further evaluations so far; in the up-right configuration flat & thin ABS substrate is required. Typically, the larger the surface, the thicker the microparticle will be, thus again undoped core and avalanching shell could partially (2 photoluminescent layers would be present) resolve the issues coming from volumetric ABS crystals; (f) self-assembled ANPs nanocrystals could be used as ABS substrate. The possible artifacts could originate from NP-to-NP variation and mostly from the quality of self-assembly to assure the homogeneity of large area ABS. LEGEND: * SEM/TEM images show the microcrystals synthesised by us, the ** comes from ref.^41^

The fabrication of thin, large, flat, and homogeneous avalanche‑backlight‑substrate (ABS) photon‑avalanching crystals could be in principle feasible using either pulsed laser deposition (PLD) or ion implantation into fluoride bulk crystals, but both approaches face significant technical challenges. PLD is a highly versatile method capable of depositing complex materials with good stoichiometry transfer, making it conceptually suitable for preparing lanthanide‑doped thin films required for photon‑avalanching functionality. However, its flexibility comes with substantial complexity: small deviations in key parameters such as laser fluence, background gas pressure, and substrate temperature can markedly affect crystallinity, uniformity, and the resulting optical properties. Achieving large‑area, flat films with homogeneous dopant distribution is particularly demanding because PLD growth dynamics are very sensitive to target composition, plume expansion, and substrate heating conditions. This sensitivity makes the controlled, reproducible fabrication of millimetre‑scale homogeneous avalanche‑active layers a non‑trivial challenge. Moreover, the underlying laser–material interaction is itself a highly complex physical process, especially when using high‑energy UV excimer lasers that strongly modify the target surface, adding additional unpredictability to film growth and microstructure.

Ion implantation into bulk undoped crystals offers a different route, enabling depth‑controlled introduction of lanthanide ions into high‑quality optical hosts. While this provides precise control over dopant placement, the process inherently introduces lattice damage requiring post‑implantation annealing. Fluoride crystals, however, are particularly sensitive to thermal and radiation‑induced defects, and fully restoring crystalline quality without inducing unwanted diffusion or clustering of dopants is difficult. For photon‑avalanching materials, which rely on finely tuned energy‑transfer pathways and highly uniform lanthanide concentration, even small local variations can suppress or destabilize the avalanche effect. Ensuring laterally homogeneous dopant distribution over large areas is another persistent challenge, as implantation profiles naturally vary with beam geometry and local crystal structure. In both fabrication routes, achieving the required combination of flatness, thickness uniformity, low defect density, and spatially uniform dopant networks represents a significant materials‑engineering obstacle.

Overall, while both PLD and ion implantation offer realistic pathways toward engineered ABS photon‑avalanching layers, the practical realization of large, homogeneous, high‑quality structures demands extensive optimization, advanced monitoring, and precise control over process conditions. The extreme sensitivity of photon‑avalanching phenomena to composition, defects, and uniformity further amplifies these challenges, making such fabrication possible but technically demanding.

## S5.4 Future directions in understanding the PA interaction with the sample

The steep relationship between pump and emission intensity, which is observed in PA materials explains the susceptibility of the photon avalanche to minute variation of excitation conditions. While challenging in terms of metrology and optical system design, when properly managed, this stand behind the concept of TAB imaging. There are a few factors that may affect the PA emission intensity, and we strongly believe that for this reason, TAB imaging can become a very versatile and sensitive tool to study not only morphology but also numerous physical, chemical and biochemical interactions at the interface of ABS crystals.

Beside simple attenuation (absorption and/or scattering) of the excitation light by the sample, which is mostly discussed at present, one cannot exclude numerous physical and chemical phenomena:

1. **Temperature quenching (TQ)**. We have previously studied, both theoretically ^38^ and experimentally ^37^ how temperature modifies the performance of avalanching materials. In both cases, upon increased temperature, the PA threshold were increased, which was directly translated (because the TAB imaging is performed at fixed pump power) to luminescence decrease. However, in the course of additional experiments (below **Figure S24**), we noted a small but systematic rise of PA intensity by 7.5 and 14% for the Au film and Au islands after 80 s illumination. While the PA intensity vary, , the susceptibility of PA to temperature shall be a simple and super-resolvable method to map the temperature distribution at nanoscale ^41–43^, if only other static interactions are excluded.
2. **A direct contact** (**n** index) related to the index of refraction change from the pure medium (air, 0.9%NaCl physiological liquid, immersion oil, etc.) to the sample (e.g. optical components, materials, cell membrane, molecules, etc.).
3. **Non-radiative resonant energy transfer** (RET)– e.g. graphene, acceptor molecules, quenchers, etc. would quench the PA emission by direct, resonant, non-radiative energy transfer^19^. Because the energy transfer is, according to Forster mechanism, limited to short <10 nm distances, this shall further enhance the contrast in super-resolution regime.
4. From the other hand, **plasmonic (P)** objects, such as Au nanoparticles are known to enhance the fluorescence by concentrating the excitation or emission field (these studies are underway) is the plasmon-emitter distance is precisely controlled. Our work on impact of plasmonic on PA emission is ongoing as a separate work, but there, c.a. 10-fold reduction of PA threshold was found. This however requires very short, single 2-5 nanometers displacement between ABS and AuNPs, otherwise local quenching (if the distance is shorter) or simple attenuation (for distances > 10nm) shall be expected. In our experimental configuration, where the ABS crystal is placed on top of is-situ formed Au Islands, we are not able to control the distance precisely.

These *TQ*, *RET*, *P* and *n* effects may occur independently or all together, but deconvolution which is present or dominating should be relatively easy^34^. To gain additional local information, one would need to perform locally, pump power dependent (PPD) luminescence intensity characterization and this is technically simple and affordable to be included during TAB imaging.

## S5.5 Future directions in optimal composition of the ABS crystals

Currently used ABS crystal contained 3% of Tm^3+^ ions, which offered the non-linearities S ~9. To further optimise the performance of TAB imaging, one should consider lower PA thresholds, higher non-linearities and larger dynamic responsivity of ABS backlight materials. Although we have studied some of these aspects ^19,44^, this still remains a challenge. In general, the balance between energy migration (EM) and cross-relaxation (CR) is required. Both, EM, CR and other energy transfer mechanisms in bulk-like heavily doped crystals are important phenomena to be evaluated and discussed in the view of possible degradation of the optical resolution of TAB imaging.

The energy migration (EM) is a known phenomenon and frequently discussed issue in heavily lanthanide doped materials, but great majority of studies are related to the energy migration occurring though Yb^3+^ ^45–50^ or sometimes through Gd^3+^ ions ^51^. In heavily Tm^3+^ avalanching materials, the EM has been discussed in a few papers only ^44^. In Stokes luminescent Ln3+ doped materials, the often used synonym of energy cross-relaxation is concentration quenching, but in PA materials CR is highly desired, because this is CR which generates energy looping, excited population multiplication and PA emission.

There are two aspects of the EM and CR that are related to the TAB imaging:

- First of all, EM should affect (in positive way) the signal strength during TAB imaging, because it will contribute to pre-pumping of the dopants and will initiate the population of the (typically empty) looping level, thus should facilitate energy looping and effectively “shorten” the PA risetimes. Ultimately, such EM should enhance photon counts at typically short dwell time per pixel.
- The second aspect is related to the impact of EM on the optical resolution of raster scanned TAB imaging. In PASSI imaging mode individual avalanching nanoparticles are imaged, however in TAB mode, the bulk ABS crystal becomes photoexcited in sequential positions across it surface. The EM in general requires an appropriate, short distance between the ions participating in energy transfer as well as long-living excited states. In case of gadolinium ions in fluoride matrices (i.e. LiYF4) the ^6^P_7/2_ excited state of gadolinium is ~ 8 ms ^52^. Energy migration in Gd^3+^ has been theoretically shown to walk over 136 nanometers ^51^. In the case of Yb3+ ions, it was found by simulations that 40% Yb doped materials can enable EM to up to 400 nm distances, which got much shorter <50 nm for 4%Yb^3+^ down to < 10 nm for as little as 0.1% of Yb^3+^. However both Gd and Yb ions have large energy gaps exceeding 35 000 and 10 000 cm^-1^, respectively and such energy levels structure is favourable for EM. However, the EM has been found to be counterbalanced (i.e. the EM distances have been shortened) by energy cross relaxation (CR) phenomenon (Fan et al., 2021). For example in Nd^3+^ ions, which encompass not only EM but are susceptible to CR, much shorter < 6 nm energy migration distances were calculated for 0.1% up to 20-25 nm for 4-40% of Nd^3+^ doping. Therefore, if Nd^3+^ were used to achieve PA (which require increased concentration of looping ions) the EM distances would be lower or similar at the worst case to the optical resolution expected from non-linear (N=20) ABS crystals. However, Tm^3+^ ions are known to be much more susceptible to concentration quenching as compared to Yb^3+^ or Nd^3+^, thus we expect, the 3% doping, used in our case, will exhibit <30 nm migration paths, thus ultimately will not affect the optical resolution.

In case of thulium ions most of the excited energy levels lifetimes is around hundreds of µs, however ^3^F_4_ energy level lifetime in 3%Tm doped LiYF_4_ reaches 13.6 ms ^19^ and energy migration occurs through energy transfer: ^3^F_4_ (Tm^3+^(1)) + ^3^H_6_( Tm^3+^(2))→ ^3^H_6_( Tm^3+^(1)) +^3^F_4_ (Tm^3+^(2)). The population of looping ^3^F_4_ excited states of thulium ions, staying outside the area of the excitation beam with a power density exceeding the threshold will thus enhance the pre-pumping state thus facilitating the looping and ESA, but this shall not affect the super-resolution.

## S5.6. General considerations related to the TAB imaging & interpretation

Despite the technical and conceptual simplicity, the interpretation of images can be more difficult, because of:

- the sensitivity of the PA signal to tiny variation of the excitation intensity. This can occur from the sample shadowing the excitation beam, but can also result from physical interaction (both quenching or enhancements), excitation light scattering on imperfections (edges, macroscopic mechanical defects). This issues should enforce the development of large, flat and homogenous ABS materials.
- The other complication with the non-linear PA substrate is the fact the luminescence rises very slowly ( tens to hundreds of milliseconds) in response to the photo-stimulation. While 100 microsecond dwell time was sufficient to image avalanching NPs in PASSI mode, in TAB configuration the same or even shorter dwell times may be expected, because a solid bulk ABS crystal is illuminated rather than the less efficient and surface quenched ANPs. The fact the scanning beam generate the strong PA emission in the exact centre, does not preclude the ‘pre-pumping’ occurring at the wings of the Gaussian beam, which facilitates the PA emission, when the excitation beams moves to a new position along its path. This inherent feature of the PA phenomenon may however complicate data analysis, because the final image will strongly change for various excitation pump intensities, dwell times and step size, which all, owing to long risetimes simultaneously affect the photon counts. This issues should enforce the precise optimisation of parameters used for imaging.
- The resolution improvements enables imaging below diffraction limit of light ~$1/\sqrt{N}$, which is however limited to a few-fold (ca.3 for N~10) improvement. The visualisation of details may however be hampered for increased distance between the sample and the ABS crystal. In current configuration of bi-pyramidal ABS such control remains difficult. This issues should enforce the development large, flat and homogenous ABS materials and up-right microscopic configurations with better control of sample deposition of the ABS crystals.
- The effective TAB contrast and resolution improvement is also pump power dependent – it remains high for pumping intensities that work below saturation region. Although larger pumping intensities will increase the photoluminescence brightness, but will reduce the non-linearities and thus will reduce the contrast and optical resolution. This issues should enforce the precise management of pumping intensities.

Further resolution enhancement can be achieved with higher non-linearities of the ABS materials, but due to very rapid intensity growth, which is typically limited to 2-3 orders before saturation occurs, the extremely high S values (S~500 were reported recently) are desirable from the optical resolution perspective, but may ultimately lead to a binary (0-1) image with very shallow dynamic range. This issues should enforce the evaluation of novel ABS materials aiming to optimize the balance between enhanced resolution and optimal dynamic range of the signal.

## S5.7. The impact of PA nonlinearity

Materials with N > 40, and in extreme cases approaching N ~ 500, have been reported (Liang et al., Nat. Nanotechnol. 17, 524 (2022); Liu et al., Nature 643, 669 (2025); Dong et al., Nat. Photonics 19, 692 (2025)). Following relationships presented in Figure 1, increased nonlinearity should improve the resolution and the contrast of TAB imaging. However, the nonlinearity index N is not the only parameter relevant for practical avalanche-substrate applications. Besides the routinely reported emission-intensity-versus-pump-power dependence, several other figures of merit must be taken into account:

•	the pump-power-dependent rise time, which affects the achievable pixel dwell time and hence imaging speed;

•	the gain, D_AV_ = I(2·I_P_)/I(I_P_), which quantifies the emission intensity rise obtained upon doubling the excitation intensity. Some materials combine a high nonlinearity index with an experimentally limited D_AV_ (< 10), because the emission profile saturates shortly beyond the avalanche onset;

•	the width of the “PA window” — defined here as I_PS_/I_TH_, the ratio between the excitation intensities corresponding to saturation (I_PS_) and to the avalanche threshold (I_TH_), respectively — which sets the excitation-power range over which a high nonlinearity is sustained. Outside this window (I_P_ < I_TH_ or I_P_ > I_PS_), N drops below 2 and provides no imaging benefit. This window get narrowed as N increases: for N ~ 500 the transition between the pre-threshold and saturated regimes becomes almost discontinuous, turning the ABS response into a binary “attenuation / no-attenuation” switch, which would be highly susceptible to artefacts (e.g. ABS inhomogeneity, sample-dependent local attenuation) and, in practice, less useful quantitatively. In addition, as N increases the 1/√N resolution-scaling function flattens, so the resolution gain per unit increase in N progressively diminishes.

From a practical standpoint, an ideal ABS material for TAB imaging would combine (i) a high nonlinearity index together with a wide dynamic range (large D_AV_ and a broad PA window), and (ii) an ideally flat, ultra-homogeneous, large-area (>100 µm) and ultrathin (<20 nm) crystal geometry. At present no such material exists: fluoride hosts are essentially the only matrices exhibiting room-temperature photon avalanche while remaining photo- and chemo-stable, non-hygroscopic and excitable with inexpensive, commercially available laser diodes, yet they are difficult to grow as thin, homogeneous layers; conversely, fabrication routes suited to thin luminescent layers (e.g. ion implantation) are not compatible with fluoride hosts.

# S6. Optical setup

## S6.1 Setup outlook

***Figure S27*** *Scheme of used optical setup.*

Spectroscopic characterization of the samples was carried out using a home-built fluorescence microscope based on the body of Nikon Ti-U Eclipse inverted microscope and shown schematically in **Figure S27**. Excitation light at 1059 nm was provided by a single mode laser module (1064CHP, TEM_00_ Gaussian mode, 3SP Technologies), and controlled and thermally stabilized by a dedicated controller (ITC4001 controller + LM14S2 butterfly mount, Thorlabs). In order to automatically and precisely regulate the power of produced beam, motorized system of neutral density filters (NDF) was utilized. First of them (NDF1) was a gradient neutral density filter (NDC-100C-4M, Thorlabs) used for gradual tuning of output power, while the other (NDF2) set of neutral density filters (FW121CNEB with preloaded ND filters, Thorlabs) was used for rough selection of the range of accessible light powers. After passing this module the beam was directed to the glass slide reflecting part of radiation to the optical power meter sensor (S121C+PM100, Thorlabs) and reference power value is monitored, which can be further used to determine the power density of the light illuminating the sample. Most of light (90%) is transmitted through the beam splitter. To overfill the back aperture of the objective, the laser beam is expanded using manual beam expander. Subsequently, the beam is directed by a dichroic mirror (DM2, DMSP900R, Thorlabs) to the lens (L) and enters the microscope’s side port. Inside the microscope, light is reflected by the mirror (M) and, after passing through the lenses (L), enters the objective lens (O, Plan Apo λ 40x, NA=0.95, Nikon, and oil immersion Plan Apo λ 100x, NA=1.45, Nikon), where it is focused on the sample. The sample stage is integrated with an xyz piezoelectric stage (PI-545 system, and E-727.3CDA controller, Physik Instrumente) for raster scanning. The same objective lens is utilized for collecting of the sample emission and directing it back through the system of mirrors (M) and Lenses (L). Next, the beam is transmitted by the DM2 and passes through a set of filter wheels consisting of neutral density filters (ND3 = OD1.0, OD2.0, or OD4.0, Thorlabs), which are utilized to limit the intensity of signals exceed the dynamic range of the detectors. Once formed and pre-filtered, the emission signal can be directed through two equal focal length bio-convex lenses and a 200 μm pinhole (P200K, Thorlabs), which is kept at the focal point. The signal then passes through a band-pass filter (BPF, FB800-40 Thorlabs) before entering the avalanche photodiode unit (APD Counts© Laser Components). The photon counts are integrated with a PCIe-6351 National Instruments PCI card, which are synchronized with piezo stage movements. Alternatively, emission light can be directed to the setup dedicated to emission spectra measurements, composed of Andor Shamrock 500i Czerny-Turner type monochromator with 3 gratings turret (GT) and equipped with the Andor Newton 920P-BEX2-DD CCD camera with active area chip cooled down to -60°C to limit the detector dark current. White light images were acquired with Andor iXion 897 512x512 EMCCD camera.

## S6.2. Modifications to confocal microscope required to adopt TAB imaging

Application of TAB imaging is indeed very simple – it basically exploits the conventional raster scanning confocal microscope. As compared to commercially available confocal microscopes, the following modifications have to be implemented to make it suitable for TAB imaging:

1. Laser source – we use a cheap, affordable, telecom wavelength pigtail laser (c.a. <6 000 Euro, e.g. [Pigtailed Distributed Bragg Reflector (DBR) Single-Frequency Lasers, Butterfly Package](https://www.thorlabs.com/newgrouppage9.cfm?objectgroup_id=7573)) working in continuous mode (typical lifetime of 10 000 hours). The controller cost is <2500 Euro (e.g. [Compact Laser Diode Driver with TEC and Mount for Butterfly Packages](https://www.thorlabs.com/newgrouppage9.cfm?objectgroup_ID=5882&pn=CLD1015))
2. The filter cube used for imaging must be replaced with new set of filters – typically a dichroic low-pass mirror with edge between NIR excitation (here 1064nm) (e.g. [Thorlabs - DMSP900R 25 x 36mm Shortpass Dichroic Mirror, 900 nm Cutoff](https://www.thorlabs.com/thorproduct.cfm?partnumber=DMSP900R)( and visible emission + one band pass filter (here 800 nm +- 30 nm, e.g. [Thorlabs - FBH800-25 Hard-Coated Bandpass Filter, Ø25 mm, CWL = 800 nm, FWHM = 25 nm](https://www.thorlabs.com/thorproduct.cfm?partnumber=FBH800-25)) – total cost < 1 000 Euro
3. The actual sample is typically placed on the glass slide either for up-right or inverted microscope stage, for TAB the only modification from this routine approach is to place the sample on non-linear, photon avalanching substrate (ABS substrate) like using the crystals presented in our work. Such crystals are not available commercially, but they are synthesized by a simple hydrothermal method and the costs of chemical substrates is < 500 Euro per batch, which contains millions of such ABS crystals. We will be happy to openly and for free provide some portion of such sample to scientists at reasonable number of requests.

All other components (like photodetectors, scanning principles, etc.) are exactly the same as in existing confocal raster-scanning microscopes.

Beside technical modification, data acquisition should be adjusted to address specific feature of the PA phenomenon – e.g. relatively long luminescence risetimes and the need to precisely control the pumping intensities (to work in the most steep part of the “S” pump power dependence below the saturation range).

## S6.3. The stability of the photoexcitation source

Another factor important for quantitative and reliable TAB imaging is the short and long term stability of the laser beam, as any tiny variations of pump intensities are translated to much larger variations in PA emission intensities.

To understand how these variability affects the PA intensities, lets consider the power law dependence of PA emission intensity (I_E_) on the pumping intensity (I_P_) with the non-linearity index equal N.

**The Dependence on Pump Intensity** $\mathbf{I}_{\mathbf{P}}$

If $I_{P}$changes by a small relative amount $\delta I_{P}/I_{P}$, the relative change in $I_{E}$ equals

$$\frac{\delta I_{E}}{I_{E}}=N\cdot\frac{\delta I_{P}}{I_{P}}$$

which means the emission intensity is **N times more sensitive** to fractional changes in pump intensity. For example for $N=8$, a **1% fluctuation in** $I_{P}$causes an **8% fluctuation in** $I_{E}$.

**Dependence on Exponent** $\mathbf{N}$

Considering the changes in $N$, one gets:

$$\frac{\partial I_{E}}{\partial N}=(I_{P})^{N}ln(I_{P})$$

thus the relative sensitivity equals:

$$\frac{\delta I_{E}}{I_{E}}=ln(I_{P})\cdot\delta N$$

Here, the **s**ensitivity to $N$depends on $ln(I_{P})$.

**A combined effect**

$$\frac{\delta I_{E}}{I_{E}}\approx N\frac{\delta I_{P}}{I_{P}}+ln(I_{P})\cdot\delta N$$

is presented in Figure S28, as response to fluctuations of emission intensity (by -20 to 20%) to pumping power and N (by -1 to +1), normalized to nominal values (N=8 and 30).

**Figure S28. The sensitivity of photoluminescence intensity to fluctuations** of (A) N (N=8 fluctuating by -1 t o+1) versus I_P_; (B) I_P_ (by -20 to +20%) versus N; (C) N (N=30 fluctuating by -1 t o+1) versus I_P_; (D) I_P_ (by -20 to +20%) versus N.

We may expect the stability of typical laser source to be below 3%. This results in $\pm3\%$relative fluctuations of PA emission for $N=N_{\mathrm{NOMINAL}}\pm1$independently from $N_{\mathrm{NOMINAL}}$. In terms of absolute emission intensity change, the $\pm3\%$fluctuations of the excitation source produce up to $\pm28\%$changes for N=8 and up to $\pm150\%$changes for N=30. Further active noise cancelling optical elements^[1]^ may be employed to reduce fluctuations of excitation source. Keeping the stability of the laser source below 0.05 % (as in device^2^) would reduce fluctuations of the absolute PA emission intensity below 4% and 1.5% respectively for N=8 and 30 (**Figure S28**).

More difficult to manage are local fluctuations of the non-linearity index, which could originate from non-perfect homogeneity of the ABS crystals. There are a few solutions: (a) further optimization of the synthesis of the ABS substrates (by stabilisation and homogenization of synthesis conditions), (b) designing different ABS’es such as self-assembled avalanching NPs (some imperfect deposition of closely spaced avalanche NP can be expected, likewise lower brightness), (c) ion implantation of avalanching ions (very costly) to create perfect ABS. The in-situ correction of the TAB images could be theoretically possible when the pristine ABS is evaluated beforehand, and then used to correct the local imperfections.

# S7. Supporting information references

1.	Biran, I., Houben, L., Kossoy, A. & Rybtchinski, B. Transmission Electron Microscopy Methodology to Analyze Polymer Structure with Submolecular Resolution. *Journal of Physical Chemistry C* **128**, 5988–5995 (2024).

2.	Gao, X. *et al.* Enhancing scanning electron microscopy imaging quality of weakly conductive samples through unsupervised learning. *Sci. Rep.* **14**, 1–11 (2024).

3.	Feng, Y. & Roos, W. H. Atomic Force Microscopy: An Introduction. *Methods in Molecular Biology* **2694**, 295–316 (2024).

4.	Perry, J. W. & Rumi, M. Two-photon absorption: an overview of measurements and principles. *Advances in Optics and Photonics, Vol. 2, Issue 4, pp. 451-518* **2**, 451–518 (2010).

5.	Silva, W. R., Graefe, C. T. & Frontiera, R. R. Toward Label-Free Super-Resolution Microscopy. *ACS Photonics* **3**, 79–86 (2016).

6.	Höppener, C. *et al.* Tip-enhanced Raman scattering. *Nature Reviews Methods Primers 2024 4:1* **4**, 47- (2024).

7.	Cheng, J. X. *et al.* Advanced vibrational microscopes for life science. *Nature Methods 2025 22:5* **22**, 912–927 (2025).

8.	Zhitnitsky, A., Benjamin, E., Bitton, O. & Oron, D. Super-resolved coherent anti-Stokes Raman scattering microscopy by coherent image scanning. *Nature Communications* **15**, (2024).

9.	Adhikari, S. *et al.* Photothermal Microscopy: Imaging the Optical Absorption of Single Nanoparticles and Single Molecules. *ACS Nano* vol. 14 Preprint at https://doi.org/10.1021/acsnano.0c07638 (2020).

10.	Wu, B. K., Tsai, S. F. & Hsieh, C. L. Simplified Interferometric Scattering Microscopy Using Low-Coherence Light for Enhanced Nanoparticle and Cellular Imaging. *Journal of Physical Chemistry C* **129**, (2025).

11.	Rogers, E. T. F. *et al.* A super-oscillatory lens optical microscope for subwavelength imaging. *Nature Materials 2012 11:5* **11**, 432–435 (2012).

12.	Zanini, G., Korobchevskaya, K., Deguchi, T., Diaspro, A. & Bianchini, P. Label-Free Optical Nanoscopy of Single-Layer Graphene. *ACS Nano* **13**, (2019).

13.	Wang, P. *et al.* Far-field imaging of non-fluorescent species with subdiffraction resolution. *Nature Photonics 2013 7:6* **7**, 449–453 (2013).

14.	Yonemaru, Y. *et al.* Super-spatial- and -spectral-resolution in vibrational imaging via saturated coherent anti-stokes Raman scattering. *Phys. Rev. Appl.* **4**, (2015).

15.	Huszka, G. & Gijs, M. A. M. Turning a normal microscope into a super-resolution instrument using a scanning microlens array. *Sci. Rep.* **8**, 1–8 (2018).

16.	LaRocca, F., DuBose, T. B., Farsiu, S. & Izatt, J. A. Optical photon reassignment super-resolved scanning laser ophthalmoscopy (Conference Presentation). *Bios* 45 (2017) doi:10.1117/12.2252384.

17.	Joseph, J., Faiz, K. P., Lahrberg, M., Tinguely, J. C. & Ahluwalia, B. S. Improving the space-bandwidth product of structured illumination microscopy using a transillumination configuration. *J. Phys. D Appl. Phys.* **53**, 044006 (2019).

18.	Stöhr, R. J. *et al.* Super-resolution fluorescence quenching microscopy of graphene. *ACS Nano* **6**, (2012).

19.	Majak, M., Misiak, M. & Bednarkiewicz, A. The mechanisms behind extreme susceptibility of photon avalanche emission to  quenching. *Materials Horizon* **11**, 4791–801 (2024).

20.	Kwon, D. The secret lives of cells - as never seen before. *Nature* **598**, 558–560 (2021).

21.	Bednarkiewicz, A., Chan, E. M.-Y. E. M. E. M., Kotulska, A. M., Marciniak, L. & Prorok, K. Photon avalanche in lanthanide doped nanoparticles for biomedical applications: super-resolution imaging. *Nanoscale Horiz.* **4**, 881–889 (2019).

22.	Denkova, D. *et al.* 3D sub-diffraction imaging in a conventional confocal configuration by exploiting super-linear emitters. *Nat. Commun.* **10**, 1–12 (2019).

23.	Plöschner, M. *et al.* Simultaneous super-linear excitation-emission and emission depletion allows imaging of upconversion nanoparticles with higher sub-diffraction resolution. *Opt. Express* **28**, 24308 (2020).

24.	Lee, C. *et al.* Giant nonlinear optical responses from photon-avalanching nanoparticles. *Nature* **589**, 230–235 (2021).

25.	Liang, Y. *et al.* Migrating photon avalanche in different emitters at the nanoscale enables 46th-order optical nonlinearity. *Nat. Nanotechnol.* **17**, 524–530 (2022).

26.	Zhu, Z. *et al.* Three-dimensional, dual-color nanoscopy enabled by migrating photon avalanches with one single low-power CW beam. *Sci. Bull. (Beijing).* **69**, 458–465 (2023).

27.	Wang, C. *et al.* Tandem Photon Avalanches for Various Nanoscale Emitters with Optical Nonlinearity up to 41st‐Order through Interfacial Energy Transfer. *Advanced Materials* **2**, e2307848 (2023).

28.	Wu, H. *et al.* Versatile cascade migrating photon avalanches for full-spectrum extremely nonlinear emissions and super-resolution microscopy. *Advanced Photonics* **6**, (2024).

29.	Dong, H. *et al.* Parallel photon avalanche nanoparticles for tunable emission and multicolour sub-diffraction microscopy. *Nature Photonics 2025 19:7* **19**, 692–700 (2025).

30.	Liu, X. *et al.* Optical nonlinearities in excess of 500 through sublattice reconstruction. *Nature* **643**, 669–674 (2025).

31.	Lee, C. *et al.* Indefinite and Bidirectional Near Infrared Nanocrystal Photoswitching. *Nature* **618**, 951–958 (2023).

32.	Liu, C. *et al.* Sub-60-nm isotropic 3D super-resolution microscopy through self-interference field excitation. *Optica* **11**, 1324 (2024).

33.	Pan, B. *et al.* Sidelobe-free deterministic 3D nanoscopy with λ/33 axial resolution. *Light Sci. Appl.* **14**, 168 (2025).

34.	Szalkowski, M. *et al.* Advances in the photon avalanche luminescence of inorganic lanthanide-doped nanomaterials. *Chem. Soc. Rev.* **54**, 983–1026 (2025).

35.	Newbury, D. E. & Ritchie, N. W. M. Performing elemental microanalysis with high accuracy and high precision by scanning electron microscopy/silicon drift detector energy-dispersive X-ray spectrometry (SEM/SDD-EDS). *J. Mater. Sci.* **50**, 493–518 (2014).

36.	Ashkin, A. & Dziedzic, J. M. Optical trapping and manipulation of single living cells using infra-red laser beams. *Berichte der Bunsengesellschaft/Physical Chemistry Chemical Physics* **93**, 254–260 (1989).

37.	Korczak, Z. *et al.* Sensitized photon avalanche nanothermometry in Pr3+ and Yb3+ co-doped NaYF4 colloidal nanoparticles. *Low Temperature Physics* **49**, 322 (2023).

38.	Szalkowski, M. *et al.* Predicting the impact of temperature dependent multi-phonon relaxation processes on the photon avalanche behavior in Tm3+: NaYF4 nanoparticles. *Optical Materials: X* **12**, 100102 (2021).

39.	Gnach, A., Lipinski, T., Bednarkiewicz, A., Rybka, J. & Capobianco, J. A. Upconverting nanoparticles: Assessing the toxicity. *Chem. Soc. Rev.* **44**, 1561–1584 (2015).

40.	Joubert, M. F. Photon avalanche upconversion in rare earth laser materials. *Opt. Mater. (Amst).* https://doi.org/10.1016/S0925-3467(98)00043-3 (1999) doi:10.1016/S0925-3467(98)00043-3.

41.	Ye, Z., Harrington, B. & Pickel, A. D. Optical super-resolution nanothermometry via stimulated emission depletion imaging of upconverting nanoparticles. *Sci. Adv.* **10**, 6268 (2024).

42.	Kilbane, J. D. *et al.* Far-field optical nanothermometry using individual sub-50 nm upconverting nanoparticles. *Nanoscale* https://doi.org/10.1039/c6nr01479h (2016) doi:10.1039/c6nr01479h.

43.	Wiwatowski, K. *et al.* Single up-conversion nanocrystal as a local temperature probe of electrically heated silver nanowire. *Nanoscale* **15**, 10614–10622 (2023).

44.	Dudek, M. *et al.* Size-Dependent Photon Avalanching in Tm3+ Doped LiYF4 Nano, Micro, and Bulk Crystals. *Adv. Opt. Mater.* **10**, 2201052 (2022).

45.	Ledoux, G. *et al.* Modeling Energy Migration for Upconversion Materials. *Journal of Physical Chemistry C* **122**, 888–893 (2017).

46.	Tu, L., Liu, X., Wu, F. & Zhang, H. Excitation energy migration dynamics in upconversion nanomaterials. *Chem. Soc. Rev.* **44**, 1331–1345 (2015).

47.	Wang, F. *et al.* Tuning upconversion through energy migration in core–shell nanoparticles. *Nat. Mater.* **10**, 968–973 (2011).

48.	Zuo, J. *et al.* Precisely Tailoring Upconversion Dynamics via Energy Migration in Core–Shell Nanostructures. *Angew. Chem. Int. Ed.* **57**, 3054–3058 (2018).

49.	Fan, J., Liang, L., Gu, Y. & Liu, X. (INVITED) Opposing effects of energy migration and cross-relaxation on surface sensitivity of lanthanide-doped nanocrystals. *Optical Materials: X* **12**, 100104 (2021).

50.	Pilch-Wrobel, A., Kotulska, A., Lahtinen, S., Soukka, T. & Bednarkiewicz, A. Engineering the compositional architecture of core-shell upconverting lanthanide-doped nanoparticles for optimal luminescent donor in resonance energy transfer : the effects of energy migration and storage. *Small* **18**, 2200464 (2022).

51.	Deng, R., Wang, J., Chen, R., Huang, W. & Liu, X. Enabling Förster Resonance Energy Transfer from Large Nanocrystals through Energy Migration. *J. Am. Chem. Soc.* **138**, 15972–15979 (2016).

52.	Misiak, M. *et al.* On the role of Gd3+ ions in enhancement of UV emission from Yb3+–Tm3+ up-converting LiYF4 nanocrystals. *Nanotechnology* **34**, 345702 (2023).

1. [Noise Eaters / Laser Amplitude Stabilizers](https://www.thorlabs.com/newgrouppage9.cfm?objectgroup_id=6107#:~:text=Utilizing%20a%20liquid%20crystal%20amplitude%20modulator%2C%20combined%20with,of%20within%200.05%25%20of%20a%20selected%20output%20power.)
